# Phospholipid:diacylglycerol acyltransferase1-overexpression stimulates lipid turnover, oil production and fitness in cold-grown plants

**DOI:** 10.1186/s12870-023-04379-5

**Published:** 2023-07-26

**Authors:** Sylwia Klińska-Bąchor, Sara Kędzierska, Kamil Demski, Antoni Banaś

**Affiliations:** 1 Intercollegiate Faculty of Biotechnology, University of Gdańsk and Medical University of Gdańsk, Gdańsk, 80-307 Poland; 2 Department of Plant Breeding, Swedish University of Agricultural Sciences, Lomma, Box 190, 234 22 Sweden

**Keywords:** Phospholipid:diacylglycerol acyltransferase, Seed yield, Lipid remodeling, Oilseed biotechnology, Lipid composition, Abiotic stress, Cold tolerance, Cold acclimation, Autophagy, Brassicaceae

## Abstract

**Background:**

Extensive population growth and climate change accelerate the search for alternative ways of plant-based biomass, biofuel and feed production. Here, we focus on hitherto unknow, new promising cold-stimulated function of phospholipid:diacylglycerol acyltransferase1 (PDAT1) – an enzyme catalyzing the last step of triacylglycerol (TAG) biosynthesis.

**Result:**

Overexpression of *AtPDAT1* boosted seed yield by 160% in Arabidopsis plants exposed to long-term cold compared to standard conditions. Such seeds increased both their weight and acyl-lipids content. This work also elucidates PDAT1’s role in leaves, which was previously unclear. Aerial parts of *AtPDAT1*-overexpressing plants were characterized by accelerated growth at early and vegetative stages of development and by biomass weighing three times more than control. Overexpression of *PDAT1* increased the expression of SUGAR-DEPENDENT1 (SDP1) TAG lipase and enhanced lipid remodeling, driving lipid turnover and influencing biomass increment. This effect was especially pronounced in cold conditions, where the elevated synergistic expression of *PDAT1* and *SDP1* resulted in double biomass increase compared to standard conditions. Elevated phospholipid remodeling also enhanced autophagy flux in *AtPDAT1*-overexpresing lines subjected to cold, despite the overall diminished autophagy intensity in cold conditions.

**Conclusions:**

Our data suggest that PDAT1 promotes greater vitality in cold-exposed plants, stimulates their longevity and boosts oilseed oil production at low temperature.

**Supplementary Information:**

The online version contains supplementary material available at 10.1186/s12870-023-04379-5.

## Introduction

Plants synthesize triacylglycerol (TAG) in endoplasmic reticulum, as a result of sequential acylation of glycerol-3-phosphate. Attachment of acyl groups is first catalyzed by the subsequent activity of glycerol-3-phospahte acyltransferase (GPAT) and acyl-CoA:lysophospatidic acid acyltransferase (LPAAT) leading first to the production of lysophosphatidic acid (LPA) and next phosphatidic acid (PA). In turn, PA is converted by phosphatidic acid phosphatase to diacylglycerol (DAG), a key intermediate for membrane and storage lipid production. The synthesized DAG can be converted into TAG by acyl-CoA:diacylglycerol acyltransferase (DGAT), for many years considered as the only enzyme responsible for the biosynthesis of storage lipids. DGAT catalyzes the transfer of an acyl group from the cytosolic acyl-CoAs pool to DAG, acylating sn-3 position [1]. Only in 2000, the existence of another TAG biosynthesis pathway, which is acyl-CoA-independent, has been uncovered. The discovered enzyme utilizes phospholipids, mainly phosphatidylcholine (PC) and phosphatidylethanolamine (PE), as a source of acyl groups, which attachment to sn-3 position of DAG it catalyzes, giving the enzyme its name: phospholipid:diacylglycerol acyltransferase – PDAT [2, 3].

So far, both enzymes’ role in plant biosynthesis of TAG has been extensively researched. TAG constitutes a major energy reservoir in some of plants’ organs. These organs often accumulate storage lipids in the form of oil bodies, which bud off of the ER outer membrane [4, 5]. TAG accounts for most of the seed lipid content, especially in oilseed plants. Contrary, in vegetative tissues TAG usually represents only a minor part of acyl lipids, although they possess a high capacity for TAG synthesis, storage, and metabolism [6]. Despite this, the TAG’s role in vegetative tissues remains elusive. The exact contribution and relationship between PDAT and DGAT in TAG production has been investigated to some extent. The role of each of the enzymes varies depending on the investigated plant species. In safflower seeds, PDAT activity has played the lead role in TAG biosynthesis, while in sunflower seeds DGAT has contributed more to TAG formation [7]. Zhang et al. [8] confirmed that these enzymes could compensate each other’s absence.

The discovery of both DGAT and PDAT has inspired many scientific efforts to design new cultivars with the desired acyl composition and elevated lipid content by overexpressing the genes encoding these enzymes. *DGAT* overexpression in *Arabidopsis thaliana* resulted in plants with larger seeds and greater oil accumulation [9]. Overexpressing *Saccharomyces cerevisiae* PDAT in the wild-type yeast has increased its TAG accumulation 1.5-times [2]. Further studies on Arabidopsis *PDAT*-overexpressing lines indicated no significant effects of this overexpression on total lipid content neither in 14-days old Arabidopsis seedlings nor in seeds of overexpressing lines [10].

Although Ståhl et al. [10] did not found any special effect of *AtPDAT1*-overexpression on lipid content and composition in tested Arabidopsis tissues they showed *PDAT* to be highly expressed not only in seeds but also in flowers, roots, and leaves. After this finding, research has begun to also focus on discovering the physiological role of PDAT enzyme in vegetative organs. The first morphological study, done by Banaś et al. [7], demonstrated that *PDAT* overexpression may increase the growth rate of *A. thaliana*, which for the first time directly suggested that this enzyme may play an essential role in plant growth and development. Only recent studies, conducted over a period of 5 years, have postulated an additional, potential role of PDAT, which may manifest in plants subjected to abiotic stress conditions [11–13].

Research done on *A.thaliana* with *AtPDAT1* overexpression and *pdat* knockout cultivated in vitro has suggested that the enzyme may be associated with thermotolerance. After exposure to stress temperature, the overexpression lines have exhibited better fitness and maintenance of pigmentation, while the knockout lines have diminished even further in size and have not retained any pigment [13]. Another study, also conducted on in vitro cultures, has indicated that *PDAT1*-mediated TAG accumulation has increased the heat resistance, while knockout lines have been more affected by heat stress, by weakening photosystem II (PSII) efficiency and lower seedling survival [11]. Low temperature leads to many serious morphological and physiological changes, e.g.: impairment of the photosystem, reduced chlorophyll content, reduced transpiration rate and damage of cellular structures. Ultimately, long-term stress leads to a reduction in biomass and yield or even to plant death [14].

Previous results have indicated a certain role of PDAT in plant growth and development and have signaled PDAT’s potential role in thermotolerance. However, the above-mentioned studies were performed mostly in in vitro conditions, which may significantly affect lipid composition and activity of enzymes involved in lipid metabolism and may not reflect true plant behavior in in vivo conditions [15]. Both the intensive search for new plant lines resistant to environmental stresses and the so-far-unknown role of PDAT enzymes in vegetative organs prompted us to conduct research in these areas. Our main goal in this study was to determine the physiological and molecular effects of *AtPDAT1*-overexpression in in vivo cultivated *A. thaliana* on plant fitness in both standard conditions and under long-term cold-stress (6 ^o^C). In both cultivations *AtPDAT1* overexpression accelerated plant growth rate and biomass content, delayed onset of senescence and elevated seed yield. These positive effects have led us to investigate parameters and processes related to lipid metabolism and autophagy. Plant autophagous response is triggered by stress conditions and may be a way for a plant to survive in adverse environmental conditions [16]. The results presented in this article further elucidate PDAT’s role in plant vegetative tissues and identify PDAT as a vector of improved plant cold-stress response.

## Results

### The effects of *AtPDAT1* overexpression on *A. thaliana* growth in standard and cold conditions

To investigate the physiological function of *At*PDAT1, two *AtPDAT1*-overexpressing lines of *A. thaliana* were first cultivated in soil at standard conditions. The initial effects of the overexpression on plant growth were noted at 3 weeks of cultivation, after, the overexpressing lines had developed stalks, while the control plants had not even developed flower buds (Fig. S1A). Mass measurements of aerial parts revealed that the overexpressing lines weighed more than control plants. The fresh and dry weight of the aerial parts increased approximately 53–69% and 64–90%, respectively, in comparison to control (Fig. S1BC).

**Fig. 1:**
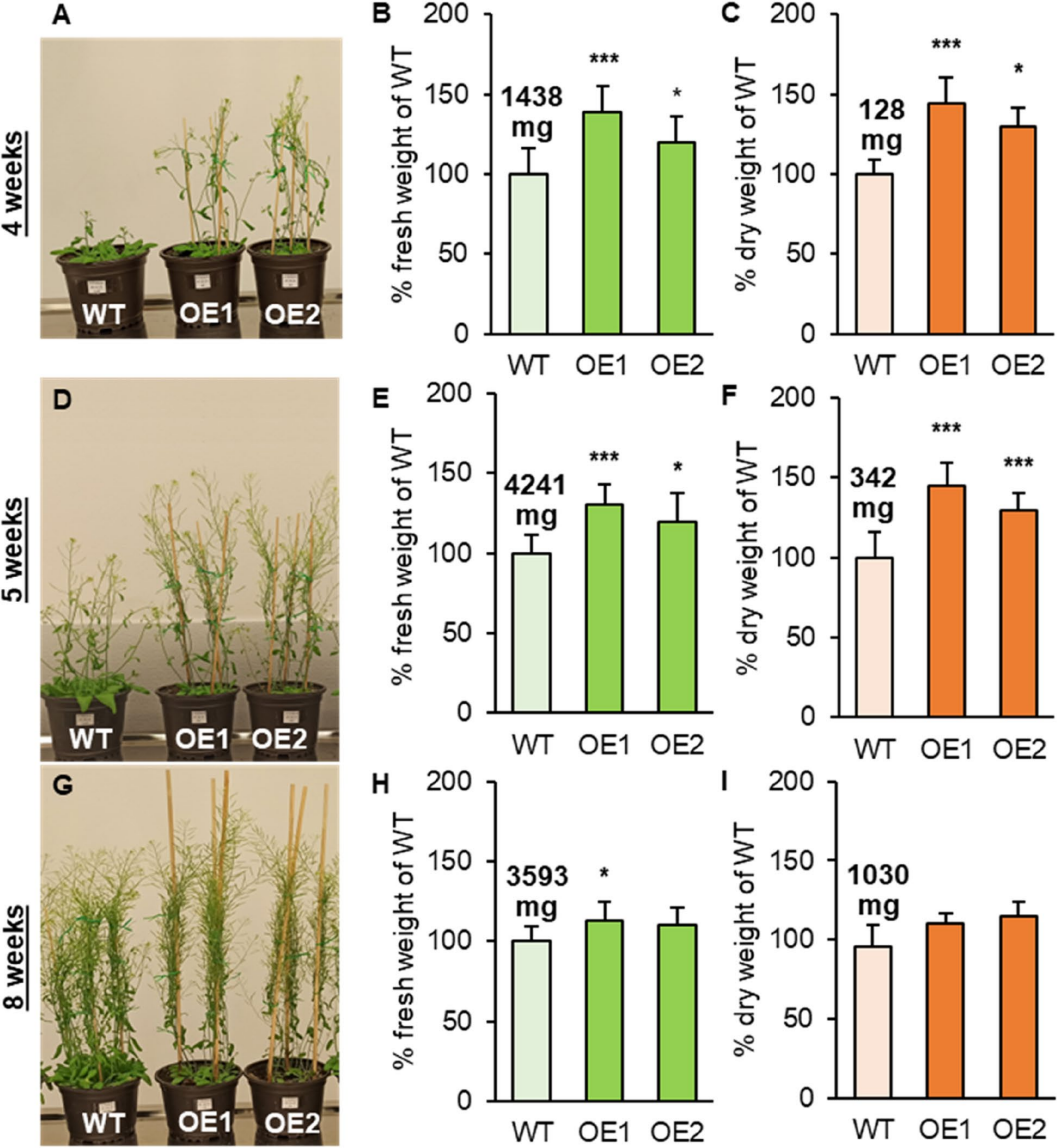
Effects of cultivation in standard conditions on wild-type Arabidopsis (WT; control plants) and *AtPDAT1 *overexpression lines (OE1 and OE2). Morphological differences between the tested lines [**A**, **D**, **G**]. Difference of the aerial parts in fresh [**B**, **E**, **H**] and dry [**C**, **F**, **I**] weight between WT and *AtPDAT1*-overexpressing lines. Results are presented as a percentage of control plants weight, with mean weight written above corresponding bars. Error bars indicate standard deviation between of at least five independent biological replicates. Asterisks demonstrate significant differences compared to WT in two-tailed Student’s t-test: (***) – *p* < 0.001; (**) –* p* < 0.01; (*) – *p* < 0.05

The next two weeks in standard conditions highlighted the observed differences with the accelerated growth rate of *AtPDAT1-*overexpressing lines, which had well-developed stalks and were blooming, while the control plants had barely formed stalks (Fig. 1AD). With the passing weeks of cultivation, the dissimilarity in growth rate and in biomass accumulation reduced (Fig. 1G). Four and five-week-old overexpressing lines increased their biomass by about 10–40% or 30–45% of fresh or dry weight, respectively (Fig. 1BCEF). The following measurement of biomass showed further reduction in the differences (Fig. 1HI). The observed accelerated growth rates are consistent with the previous observation done by Banaś et al. [7].

Second batch of plants grown for the first three weeks in standard conditions was transferred to growth chambers with temperature set to 6 ^o^C, where they were left until the end of development cycle – on average about 30 weeks after sowing. All plants grew slower and each stage of their development was extended. Long-term cold stress intensified morphological effects observed in standard conditions.

**Fig. 2:**
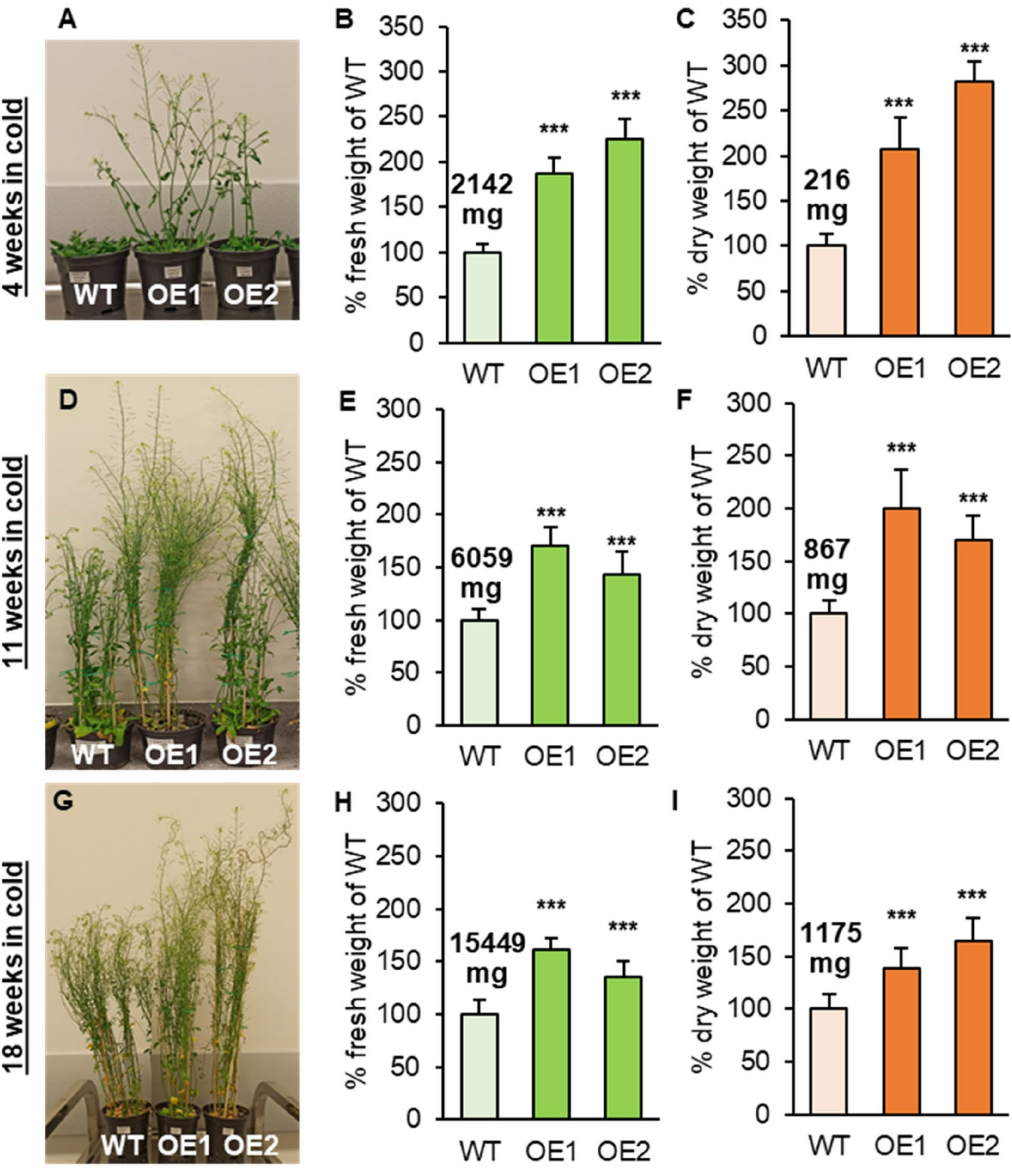
Effects of cultivation in cold conditions on wild-type Arabidopsis (WT; control plants) and *AtPDAT1* overexpression lines (OE1 and OE2); transfer to cold (6 ^o^C) after 3 weeks of cultivation in standard conditions. Morphological differences between the tested lines [**A**, **D**, **G**]. Difference of the aerial parts in fresh [**B**, **E**, **H**] and dry [**C**, **F**, **I**] weight between WT and *AtPDAT1*-overexpressing lines. Results are presented as a percentage of control plants weight, with mean weight written above corresponding bars. Error bars indicate standard deviation between of at least five independent biological replicates. Asterisks demonstrate significant differences compared to WT in two-tailed Student’s t-test: (***) – *p* < 0.001; (**) – *p* < 0.01; (*) – *p* < 0.05

After 4 weeks of cold cultivation, the fresh weight per plant of both *AtPDAT1*-overexpressing lines was approximately 3,900 mg and 4,800 mg, while the dry mass was 450 mg and 611 mg, for OE1 and OE2. At that time, the aerial part of control lines weighed about 2 or 2.5 times less, respectively compared to OE1 and OE2, and had just emerging stalks with single flowers, while the OE lines had well-developed stalks with side stems and siliques (Fig. 2A-C). The morphological changes persisted over the following weeks (Fig. 2DG). After another 7 weeks of cultivation in cold, the mass of control plants increased 3 and 4-imes for fresh and dry mass, respectively. The overexpressing lines exhibited higher accumulation of biomass at the level of about 43–70% and 70–100% higher than the fresh and dry weight of control plants (Fig. 2EF). The control plants at 18 weeks of cold exposure weighed at least 4.2 times more in fresh biomass than the same line cultivated in standard conditions at the last fresh weight measurement point (before the end of development cycle), while the dry weight increased 1.2 times (Fig. 1 HI; Fig. 2HI). The differences in weight of the aerial parts continued on until full plant maturity – the OE1 and OE2 plants weighed about 24% and 32% more than the control in cold cultivation and about 20% and 25% more in standard conditions. The cold-grown control plants weighed also almost twice as much as control plants of standard conditions in dry weight (Supplemental Fig. S2).

### Effects of *AtPDAT1* overexpression on the yield and on the acyl-lipid content and composition

The phenotypic analysis and measurement of biomass described above, showed that the *AtPDAT1*-overexpressing lines were characterized by an accelerated growth rate and a higher increase of biomass compared to the control plants. These differences were clearly visible both in standard and in long-term cold stress conditions. The next step of the research was to investigate how *AtPDAT1* overexpression and stress conditions affect the seed yield of plants and their oil quality.

**Fig. 3:**
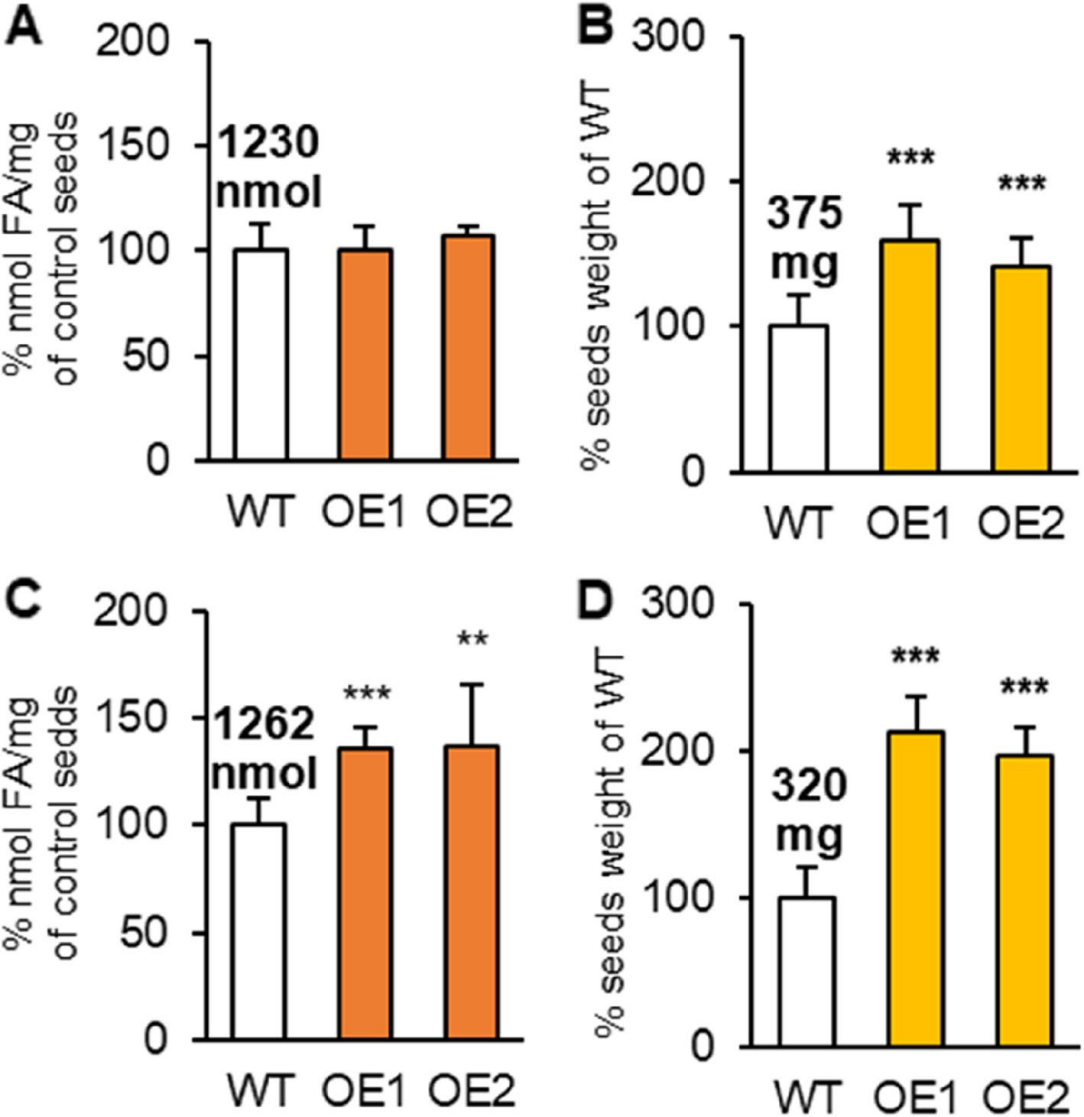
Effects of cultivation type on wild-type Arabidopsis (WT; control plants) and *AtPDAT1* overexpression lines (OE1 and OE2) acyl-lipid content and seed yield. Charts **A** and **B** present acyl-lipid content and seed yield, respectively, for plants cultivated in standard conditions. Chart **C** presents acyl-lipid and chart **D** presents seed yield from plants subjected to long-term cold stress, respectively. Results are presented as a percentage of the particular value observed in control plants. Mean values/plant are written above corresponding bars of the control. Error bars indicate standard deviation between of at least five independent biological replicates. Asterisks demonstrate significant differences compared to WT in two-tailed Student’s t-test: (***) – p < 0.001; (**) – *p* < 0.01; (*) –*p*  < 0.05

In case of standard cultivation, *PDAT* expression level did not affect oil content of the seeds. All lines accumulated approximately 1230 nmol of FA per mg of seeds (Fig. 3A). We noted, however, a small change in the composition of fatty acids in acyl-lipids of *AtPDAT1*-overexpressing lines, mainly characterized by elevated levels of gondoic acid and ‘other’ fatty acids (which include very long chain fatty acids, with more than 20 C) compared to the control; (Supplemental Fig. S3A). Previous studies support these results, neither changes in oil content nor in composition of fatty acids between control and overexpression lines were reported [10]. Despite the lack of difference in oil content, *AtPDAT1* overexpression led to greater yields, 59% and 42% higher for OE1 and OE2 lines, respectively (Fig. 3B).

This marks the first time that effects of long-term cold stress were examined for these lines. The results showed that the oil content of the stressed control plants did not differ significantly from the control plants grown in standard conditions. Nevertheless, the seeds of the *AtPDAT1-*overexpressing lines presented an elevated acyl-lipid content by about 36% in comparison to the control, but their fatty acid composition remained unchanged both compared to the control form standard and stress conditions (Fig. 3C; Supplemental S3B). Furthermore, despite a slightly reduced seed yield (statistically insignificant) of control lines in cold conditions compared to standard conditions, the seed yield of cold-grown OE1 and OE2 was remarkably greater (compared to the control) by 113% and 98%, respectively. Compared to the yield collected from OE1 and OE2 after standard cultivation, the yield of these lines in cold condition was about 16% and 19% higher, respectively (Fig. 3D). Examination of seed viability showed that seeds collected from plants subjected to long-cold stress were able to germinate, and previously noted accelerated development of overexpressing lines was still maintained (Supplemental Fig. S4).

### Impact of *AtPDAT1* overexpression and cold conditions on the lipid metabolism pathways in leaves

**Fig. 4:**
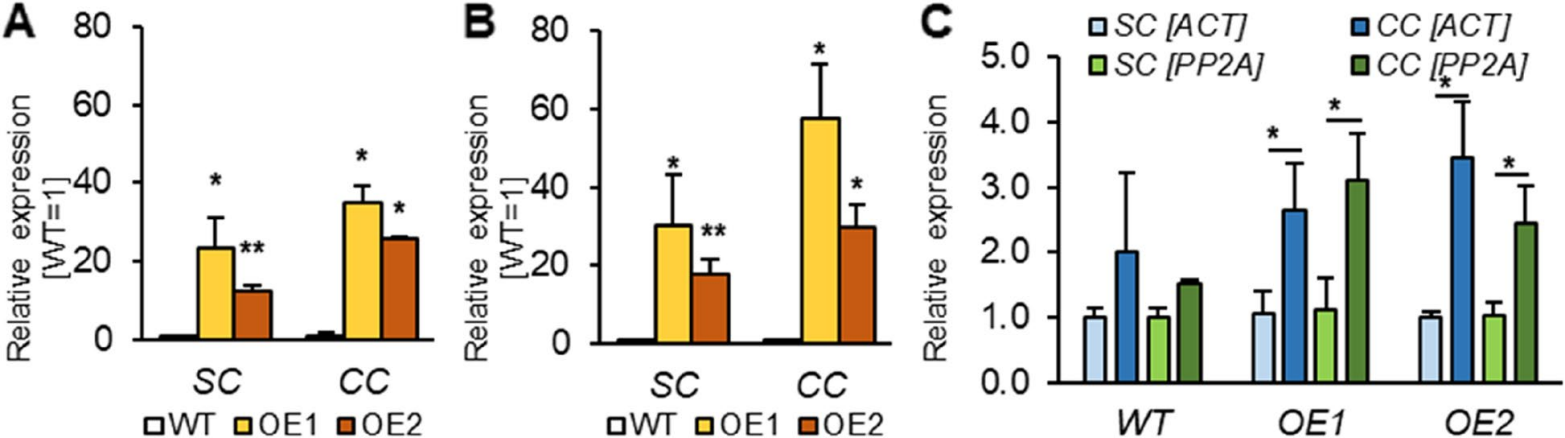
Relative expression of *AtPDAT1* in leaves of Arabidopsis wild-type (WT; control) and *AtPDAT1* overexpression lines (OE1 and OE2). The first two plots represent the expression level normalized to two housekeeping genes - *ACT* [**A**] and *PP2A* [**B**]. Presented values show the expression levels in standard conditions (SC) and cold conditions (CC) in comparison to the expression level of the control plant grown in corresponding conditions. Chart **C** represents expression levels of *AtPDAT1* among individual lines depending on cultivation conditions. Expression in wild-type line cultivated in standard conditions was used as a control. Mean values are presented on charts and error bars indicate standard deviation between three independent biological replicates. Asterisks demonstrate significant differences compared to WT [on charts A and B] or between tested cultivation conditions among one line [C] in two-tailed Student’s t-test: (***) – *p *< 0.001; (**) – *p *< 0.01; (*) – *p* < 0.05

The *AtPDAT1*-overexpressing lines used in the study exhibited an extremely elevated expression levels of *AtPDAT1*. *PDAT1* was expressed more than 24 or 30 and 13 or 18 times when plants were cultivated in standard conditions (respectively for OE1 and OE2 with reference to *ACT* and *PP2A*). Previously, a higher activity of this enzyme was also confirmed in the leaves of both tested lines, however, the increase of this activity did not affect neither polar lipid nor TAG content or composition [7, 10, 13]. The exposure to long-term cold stress almost doubled the level of *PDAT1* expression in overexpressing lines (Fig. 4AB). Comparison of the relative expression levels between the same lines cultivated in different conditions showed an elevated expression of *PDAT1* in control plants subjected to cold, indicating that the stress conditions themselves affected *PDAT1* expression level. These results are consistent with previous studies on the effects of heat on *PDAT1* expression in Arabidopsis [13] and on increased expression of *PDAT* genes in cold-treated *Camelina sativa* [12]. Our stress studies also led to an almost 3-fold increase in the expression levels of *PDAT1* gene in OE lines subjected to cold compared to these lines cultivated in standard conditions, showing that stress conditions magnify observed expression level even in OE lines (Fig. 4C). In terms of the lipid profile of plants subjected to cold, it turned out that the leaves of overexpressing lines had a slightly reduced content of polar lipids, without any changes in the content of individual classes, while TAG pools were slightly higher without statistical significance (Supplemental Fig. S5 and Fig. S6).

In spite of no changes in the polar lipid profiles of the leaves of plants cultivated both in standard and cold conditions, the assays testing the activity of two enzymes participating in phospholipid remodeling revealed their elevated activity. Both LPCAT and LPEAT enzymes belong to the group of acyl-CoA:lysophospholipid acyltransferases (LPLAT) and are mainly responsible for the modification of phosphatidylcholine and phosphatidylethanolamine, respectively. Both phospholipids play an important role as acyl donors in the PDAT reaction [10]. Additionally, lysophospholipids, formed as a by-product of PDAT activity during the transfer of acyl groups from phospholipids to DAG, constitute the main substrate for the LPLAT enzymes, thus participating in lipid turnover.

In order to measure their activity in forward reaction the in vitro radioisotope assays were performed using [^14^C]18:2-CoA as an acyl donor, which was confirmed to be one of the substrates most efficiently utilized by LPCAT and LPEAT enzymes present in Arabidopsis [17, 18]. Each assay was conducted with a previously optimized reaction time and amount of microsomal fraction, determined using the microsomal fraction derived from cold conditions (Supplemental Fig. S 7). The obtained activity is presented as the amount of the *de novo* synthesized product (corresponding phospholipid) during 1 h of the reaction catalyzed by the enzymes present in aliquots of microsomal fractions containing 1 nmol of microsomal PC.

**Fig. 5:**
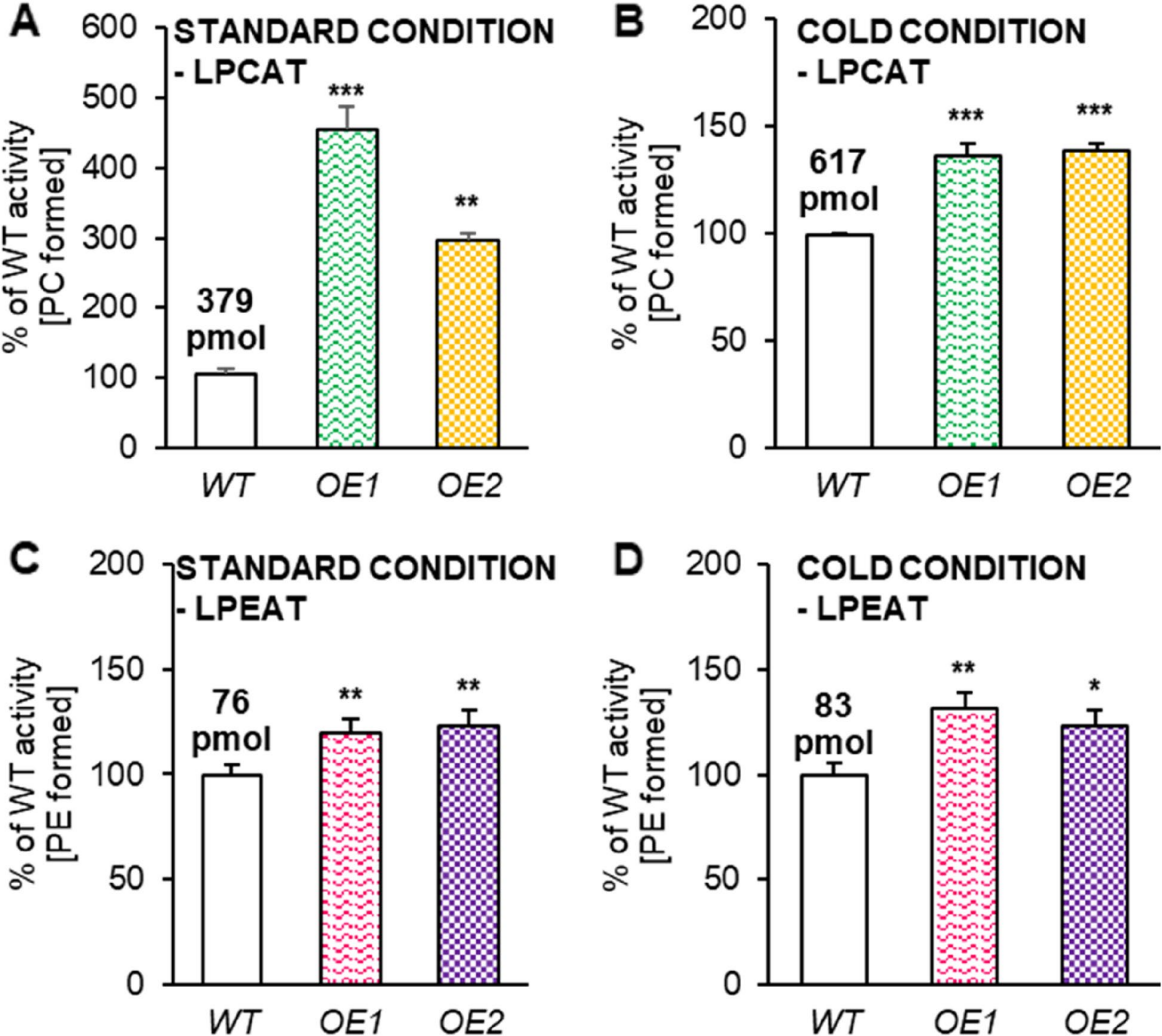
Endogenous activity of acyl-CoA:lysophospholipid acyltransferases tested in vitro in microsomal fractions isolated from Arabidopsis wild-type (WT; control) and *AtPDAT1* overexpression lines’ leaves (OE1 and OE2). Panels Charts **A** and **C** indicate the effects of overexpression in standard conditions and panels Charts **B** and **D** effects of overexpression in cold conditions on LPCAT and LPEAT activity, respectively. Results are presented as a percentage of control plants’ activity measured as pmol of [^14^C] enzymatic products (PC for LPCAT or PE for LPEAT) synthetized during 1 h reaction in the presence of aliquots of microsomal fractions containing 1 nmol of microsomal PC. The mean value is written above corresponding bars. Error bars indicate standard deviation between three replicates. Asterisks demonstrate significant differences compared to WT in two-tailed Student’s t-test: (***) – *p* < 0.001; (**) – *p* < 0.01; (*) – *p* < 0.05

From two tested acyltransferases, LPCAT enzymes turned out to be more active than LPEAT enzymes in the microsomal fractions from the control and *AtPDAT1*-overexpressing lines, regardless of the growing conditions of the plants from which they were isolated. LPCAT was the most active in overexpressing lines cultivated in standard conditions; 4.5 times and 3 times of control activity, respectively for OE1 and OE2. Cold treatment escalated LPCAT activity in control plants about 1.6 times compared to standard cultivation, however, such enhancement of LPCAT activity was not observed for *AtPDAT1*-overexpressing lines. The activity of LPCAT in microsomal fractions from leaves of both overexpressing lines cultivated in cold temperature was lower than in microsomal fractions from leaves of these overexpressing lines cultivated in standard conditions. However, the LPCAT activity in both OE lines cultivated in cold conditions was still about 37% higher than in control plants (Fig. 5AB). The activity of examined LPEAT was also elevated in the microsomal fraction of *AtPDAT1-*overexpressing lines both from standard and cold conditions by approximately 20–30%. The basal activity of LPEAT in the control lines were slightly elevated in stress conditions (Fig. 5CD).

Considering that lipid remodeling is a crucial process e.g. during regulating structure of membrane, cellular homeostasis, signaling and stress tolerance [19], the efficiency of this process was measured. Both tested LPLAT enzymes participate not only in phospholipid biosynthesis via their forward activity (LPL formed in other reaction e.g. catalyzed by PDAT or phospholipase A_2_), but also conduct a reverse-type reaction. In this type of reaction, LPLAT produce and supplement a plant cell with modified acyl-CoA and lysophospholipid pools, which can be reutilized in forward reactions. The assays were carried out with the addition of coenzyme A and BSA to shift the equilibrium of LPLAT reaction into their reverse activity (with participation of parallel PDAT and PLA_2_ activities). The results were presented as the amount of the corresponding phospholipid synthesized from *de novo* created LPL pool (products of LPLAT enzymes activity) during 1 h of the reaction and in the presence of aliquots of the microsomal fractions containing 1 nmol of endogenous PC.

Due to the previously confirmed elevated activity of PDAT in the tested overexpressing lines and the higher LPLAT activity demonstrated in this study, we assumed that these lines might have an improved remodeling process. The remodeling efficiency turned out to be the highest in case of the PC pool. Particularly high activity of this process was noticed for *AtPDAT1*-overexpressing lines grown in standard conditions: it was 1.8 times more intense for OE1 and 2.3 more intense for OE2 than for the control lines. Long-term cold conditions slowed down the remodeling process, retaining only about 15% of its activity detected for the microsomal fractions isolated from leaves of plants grown in standard conditions. Nevertheless, in case of the overexpressing lines, PC remodeling was still about 1.5 times more efficient. For the PE pool, the differences in remodeling between control and tested overexpressing lines were smaller than in the case of PC. In standard cultivation, the OE1 and OE2 lines were able to perform a more intensive fatty acid exchange in PE by about 31–34% compared to the control. In case of cold-treated PE, the lipid remodeling was similar in both control and OE microsomal fractions. However, the remodeling of PE in control microsomal fractions obtained from leaves of plants grown in cold conditions was extremely low (about 14 times lower than observed in microsomal fractions of leaves from standard conditions), (Table 1).

**Fig. 6:**
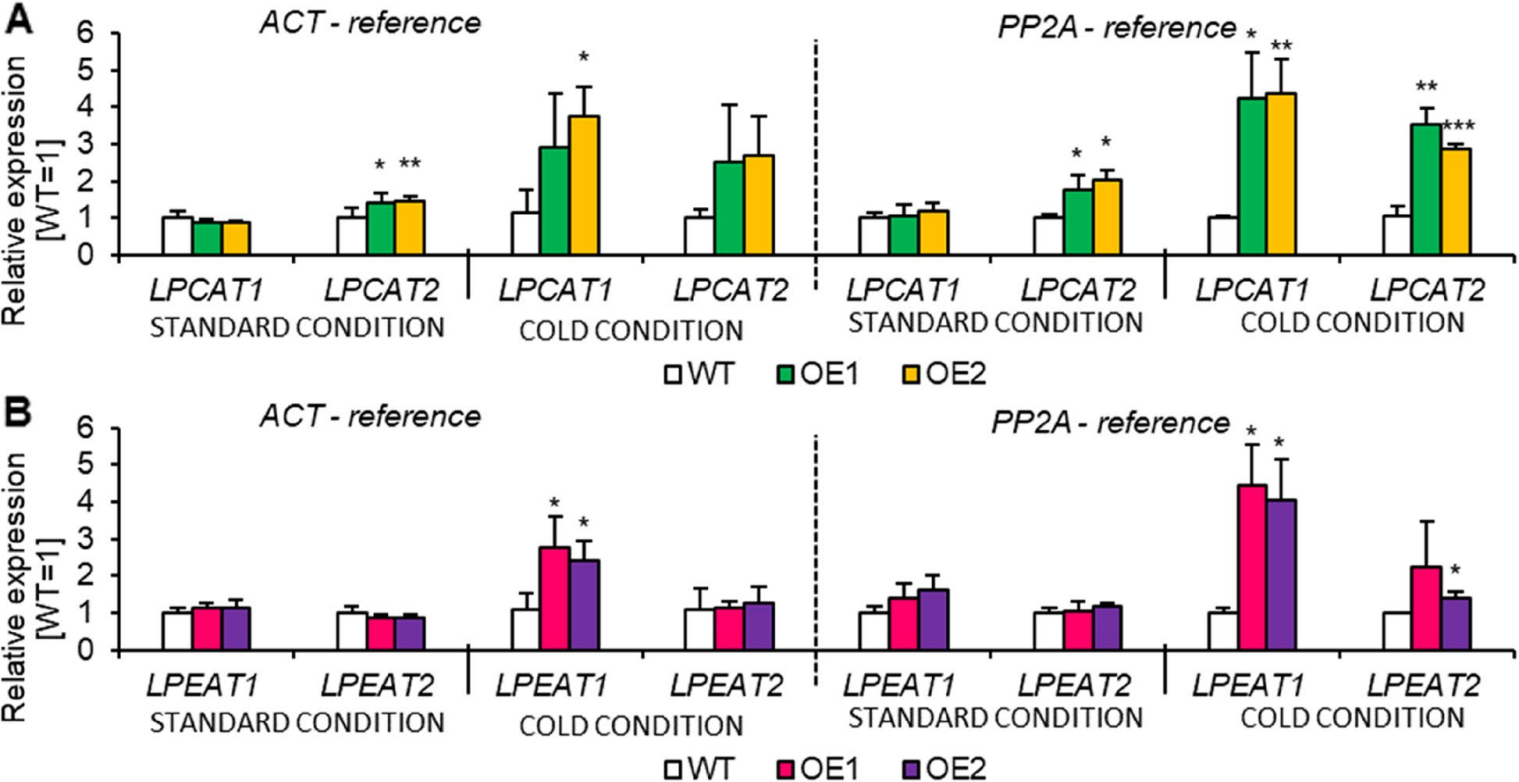
The levels of expression of genes encoding acyl-CoA:lysophospholipid acyltransferases responsible for the biosynthesis of phosphatidylcholine [**A**] and phosphatidylethanolamine [ **B**] in Arabidopsis leaves of wild-type (WT; control) and *AtPDAT1* overexpression lines (OE1 and OE2) cultivated in standard and cold conditions. Chart A indicated the expression levels of *LPCAT1* and *LPCAT2* and chart B represents the expression levels of *LPEAT1* and *LPEAT2*; normalized to *ACT* and *PP2A* housekeeping genes. Expression in control line cultivated in standard conditions was used as a control. Mean values are presented on charts and error bars indicate standard deviations between three independent replicates. Asterisks demonstrate significant differences compared to WT in two-tailed Student’s t-test: (***) – *p* < 0.001; (**) – *p* < 0.01; (*) – *p* < 0.05

Knowing that *PDAT1* overexpression may affect not only the activity of PDAT1 itself, but also the activity of other proteins involved in the lipid metabolism pathways, we decided to check possible changes at the gene expression level. For this purpose, we conducted a relative expression analysis for two LPCAT and two LPEAT isoenzymes (correspondingly *LPCAT1*, *LPCAT2* and *LPEAT1, LPEAT2*). First, we measured the relative expression of these genes in leaves of plants from standard conditions, which showed that among *LPCATs* only *LPCAT2* expression increased in *AtPDAT1*-overexpressing lines. Among *LPEATs*, minor differences between the OE and control lines were found for *LPEAT1*, however they were not statistically significant. In case of cold conditions, significant increase of the relative expression of three out of four tested genes was observed. Only the expression level of *LPEAT2* remained at the same level. However, *LPEAT1* expression *was* elevated about 2.8 or 4.4-times for OE1 and about 2.4 or 4.1-times for OE2 in reference to *ACT* and *PP2A*. Among *LPCAT*, in stressed conditions, the expression level of *LPCAT1* substantially increased by 3-times and 3.8-times for OE1 and OE2, when *ACT* was used as the reference gene. These tendencies reoccurred in reference to *PP2A*. However, *LPCAT2* expression was significantly higher only with one of the housekeeping-genes - *PP2A* (Fig. 6).

**Table 1:** The remodeling intensity of phosphatidylcholine and phosphatidylethanolamine present in leaves of Arabidopsis wild-type (WT; control) and *AtPDAT1* overexpression lines (OE1 and OE2) cultivated in standard and cold conditions. The intensity is indicated as [^14^C]acyl group incorporation from [^14^ C]18:1-CoA into PC or PE present in the microsomal fractions isolated from leaves. Mean values and standard deviations are presented in the table. Asterisks demonstrate significant differences compared to WT in two-tailed Student’s t-test: (***) – *p* < 0.001; (**) – *p* < 0.01; (*) – *p* < 0.05

TESTED LINES	Standard condition		Cold condition	
	**Phosphatidylcholine** [pmol [^14^ C]PC/min/nmol microsomal PC]	**Phosphatidylethanolamin** [pmol [^14^ C]PE/min/nmol microsomal PC]	**Phosphatidylcholine** [pmol [^14^ C]PC/min/nmol microsomal PC]	**Phosphatidylethanolamin** [pmol [^14^ C]PE/min/nmol microsomal PC]
**WT**	0.191 ± 0.02	0.098 ± 0.01	0.028 ± 0.0002	0.02 ± 0.004
**OE1**	0.348*** ± 0.004	0.132** ± 0.01	0.046* ± 0.009	0.015 ± 0.0005
**OE2**	0.447*** ± 0.03	0.129* ± 0.01	0.036* ± 0.003	0.019 ± 0.003

### Effect of *AtPDAT1* overexpression on SDP1 lipase hydrolyzing the triacylglycerol pool

This study, along with the previous ones, established that PDAT activity and expression was increased in the *AtPDAT1*-overexpressing lines. Despite this, no significant differences in TAG content were observed in standard conditions [10]. We detected a similar lack of statistically significant changes in TAG content in leaves of these lines cultivated in cold conditions (Supplemental Fig. S 5). We started to speculate that these differences between the TAG content and the increased activity of PDAT enzyme may be the result of synergistically elevated activity of TAG lipases. In Arabidopsis, there are several putative candidates for TAG lipase: SDP1 (Sugar-dependent1), SDP1L (Sugar-dependent1-like), ATGLL (Adipose Triglyceride Lipase-Like) and CGI58L (a member of the α/β-hydrolase family of proteins); [20, 21]. Study done by Fan et al. [22] revealed that SDP1 is responsible for most of the hydrolysis of the TAG pool presents in leaves.

**Fig. 7:**
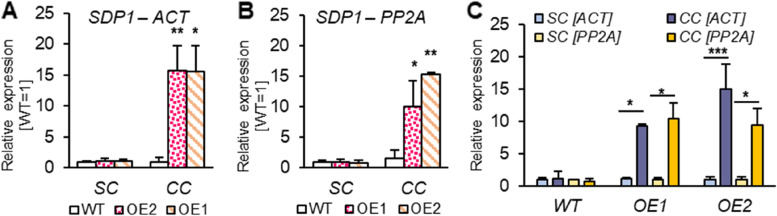
Relative expression of *AtSDP1* in Arabidopsis leaves of wild-type (WT; control) and *AtPDAT1* overexpression lines (OE1 and OE2). First two charts represent expression levels normalized to two housekeeping genes - *ACT* [**A**] and PP2A [**B**]. Presented values show expression levels in standard conditions (SC) and cold conditions (CC) regarding the expression levels of the control plants grown in corresponding conditions. Chart **C** represents expression levels of *AtSDP1* regarding just individual lines depended on cultivation conditions. Expression of corresponding lines cultivated in standard conditions were used as a control. Mean values are presented on charts and error bars indicate standard deviations between three independent biological replicates. Asterisks demonstrate significant differences compared to WT [on charts A and B] or between tested cultivation condition among one line [C] in two-tailed Student’s t-test: (***) – *p* < 0.001; (**) – *p* < 0.01; (*) – *p *< 0.05

To investigate the effect of *AtPDAT1* overexpression and cold treatment on the turnover of the TAG pool, we conducted an analysis of relative expression of the gene encoding *SDP1*. First, we measured expression in the leaves of the plants grown in standard conditions. Expression of this gene in *AtPDAT1*-overexpressing lines was only slightly. Analysis of these lines exposed to cold, showed an extremely increased expression levels of *SDP1* in OE lines: by about 16 and 10-fold for OE1 and by about 16 and 15-fold for OE2 with *ACT2* or *PP2A* used as a reference, respectively (Fig. 7AB). What is more, exposure to low temperature stimulated at least a 10-fold increase in *SDP1* expression in both OE lines. Such observations were not made for control plants (Fig. 7C).

### Effect of overexpression of *AtPDAT1* on aging and autophagy intensity in Arabidopsis plants subjected to standard and stress conditions

Previous observations of the life cycle of *AtPDAT1*-overexpressing lines for the first time revealed that mutant lines are characterized by delayed yellow leaf appearance, indicating postponed senescence [7, 13]. These observations were done for plants grown in standard conditions and they correlate with our recent findings. In this study, we assessed crucial points of plant life cycle for WT and *AtPDAT1*-overexpressing lines exposed to cold. The appearance of flower buds for all tested lines coincided with previous data and took place before the start of stress treatment for OE lines and during stress treatment for control plants. Further development of control plants was significantly postponed in long-term cold stress, as already described above (Fig. 2). The first flower appeared approximately 20 days later for the control plants compared to OE lines (Table 2). However, in OE lines the first yellow leaves appeared about 15 and 17 days later for OE1 and OE2, respectively compared with control plants (Table 2). These results indicated that in *AtPDAT1*-overexpressing lines cold conditions might have prevented senescence even more than in standard conditions and thus have promoted plant longevity. To verify if this observation could be connected with the intensity of autophagy process, we decided to analyze the effectiveness of the autophagy in these lines exposed to cold.

Autophagy is closely related to plant lipids [23]. One of the phospholipids essential for this process is PE, known to be responsible for Atg8 recruitment (an autophagy marker) and phagophore formation [23, 24]. Despite the lack of significant changes in PE content in the leaves of plants subjected to cold, a higher activity of LPEAT, re-synthesizing this phospholipid from LPE and acylo-CoA, was detected in OE lines (Fig. 5). In this study we determined the level of the marker proteins: the above-mentioned Atg8 (Atg8a) and NBR1, which acts as a cargo receptor. The main difference in the use of these proteins during autophagy is that Atg8 is deconjugated from PE in outer membrane of autophagosome, while NBR1 is degraded [25].

**Fig. 8:**
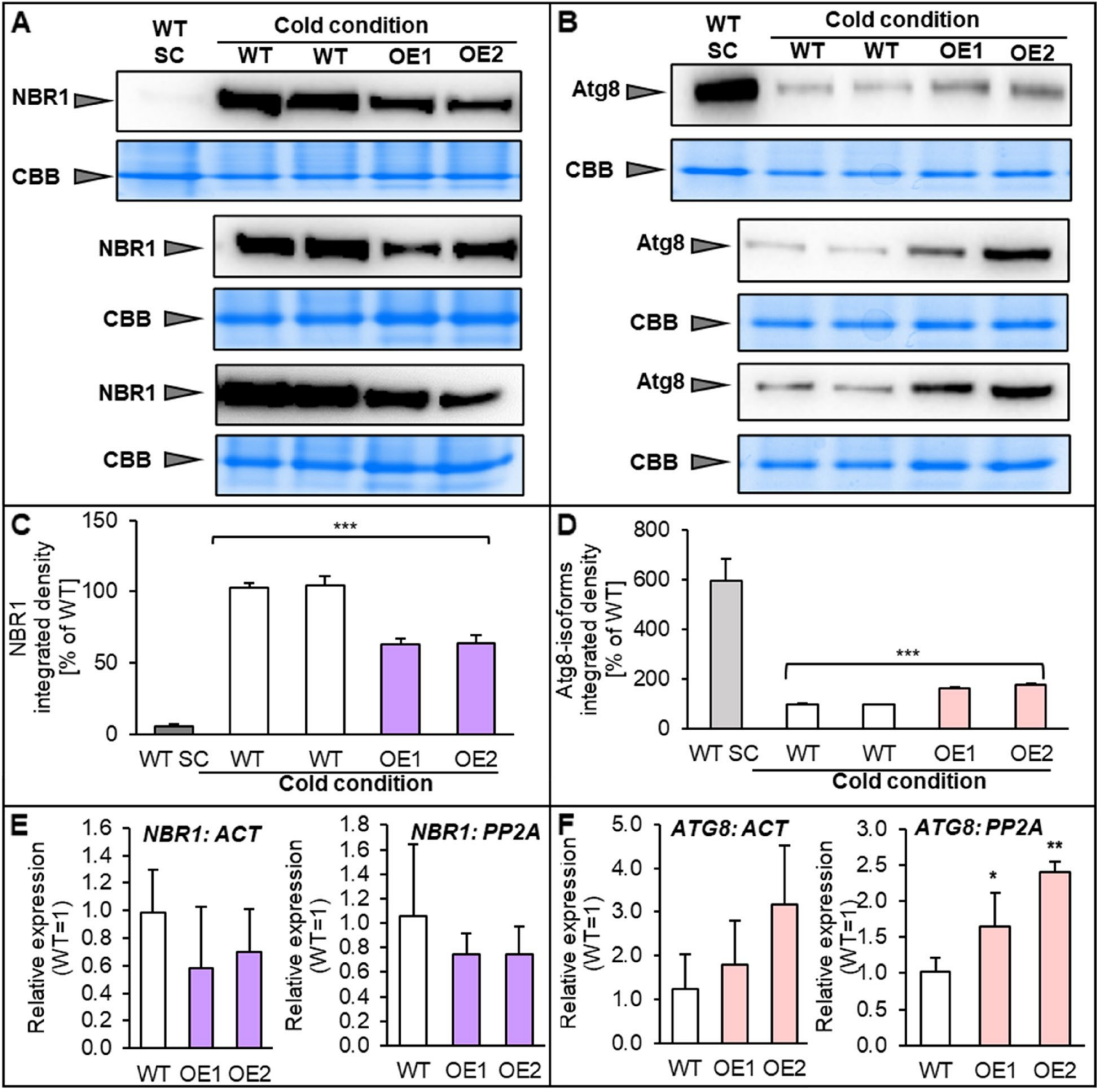
Effect of cold conditions on autophagic flux in Arabidopsis wild-type (WT; control plants) and *AtPDAT1 *overexpression lines (OE1 and OE2). Immunoblots (three replications) showing endogenous NBR1 levels [**A**] and Atg8a levels [**B**] after incubation with anti-NBR1 and anti-Atg8 antibodies, respectively, in leaves of plants cultivated 4 weeks in cold (preceded by 3 weeks in standard conditions) and in leaves of 4-week-old WT plants cultivated in standard conditions (SC). Coomassie blue staining was used as a loading control. Graphs below show quantification of bands intensity [**C** – NBR1 and **D** – Atg8a]. Results are presented as a percentage of the value in control plants (WT) cultivated in cold conditions. Chart **E** and **F** represent relative expression levels of genes encoding NBR1 and ATG8a, respectively in cold conditions. Results were normalized to *ACT* and *PP2A* housekeeping genes. Above data are presented as means and standard deviations from at least three replicates. Asterisks demonstrate significant differences compared to WT in two-tailed Student’s t-test: (***) – *p* < 0.001; (**) – *p* < 0.01; (*) – *p *< 0.05. Original western blots and stained gels are shown in the Fig. S9 and Fig. S10

**Fig. 9:**
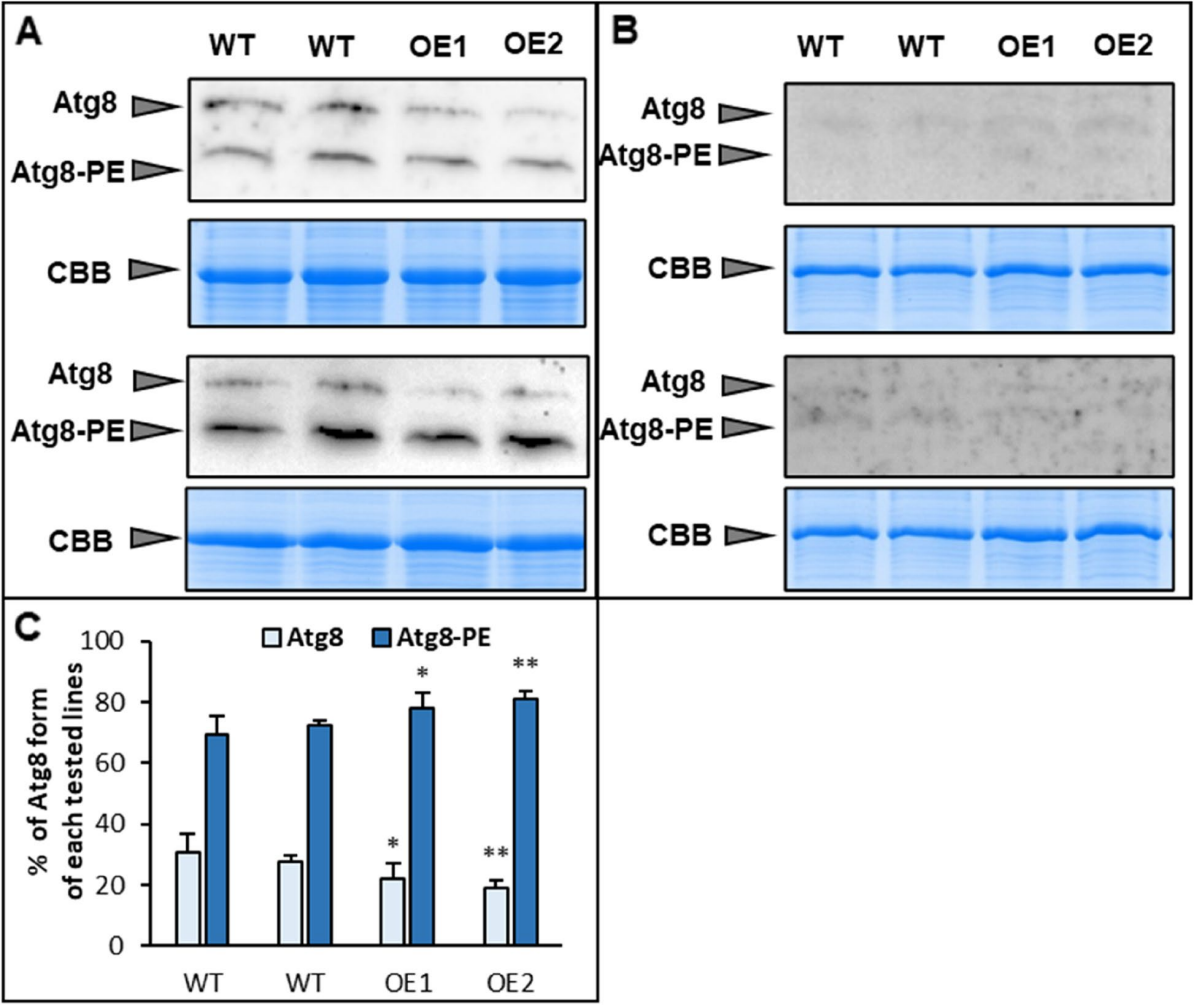
Lipidation of Atg8 present in Arabidopsis wild-type (WT; control plants) and *AtPDAT1 *overexpression lines (OE1 and OE2) cultivated in standard [**A**, **C**] and cold [**B**] conditions. Immunoblots (two selected replications) show endogenous levels of Atg8 free form and lipidated Atg8 conjugated with phosphatidylethanolamine (PE) in leaves of plants cultivated for 4 weeks in standard conditions [A] and for 4 weeks in cold conditions, preceded by 3-week standard cultivation [**B**]. Coomassie blue staining was used as a loading control. Graph **C** presents percentage of lipidated and free form of Atg8 in each line. Data are presented as means and standard deviations from at least three replicates. Asterisks demonstrate significant differences compared to WT in two-tailed Student’s t-test: (***) – *p *< 0.001; (**) – *p* < 0.01; (*) – *p *< 0.05. Original western blots and stained gels are shown in the Fig. S11 and Fig. S12

The levels of NBR1 protein in leaves of cold stressed plants of tested Arabidopsis lines turned out to be extremely increased compared to control plants grown in standard conditions, indicating a reduction, or weakening of the autophagy process under cold stress conditions. Nevertheless, compared to control line, a decreased level of NBR1 protein was detected in the *AtPDAT1*-overexpressing lines. The total amount of this protein was diminished by about 40% for both OE lines (Fig. 8AC). Analysis of relative expression of the NBR1-encoding gene showed statistically insignificant differences (Fig. 8E). Similar observations indicating impaired autophagy in cold conditions were obtained when total Atg8 protein level was examined by separation of protein extracts by NuPAGE gel without reduction. In the case of non-stressed control plants, Atg8 isoforms were 6-times more abundant compared to cold conditions (Fig. 8BD). Among the tested lines subjected to cold, *AtPDAT1*-overexpressing lines again revealed greater efficiency of autophagy as indicated by elevated level of total ATG8 proteins by approximately 62% and 78% in OE1 and OE2, respectively (Fig. 8BD). Observed changes in this protein’s content were consistent with increased expression of the gene encoding ATG8a, which was on average twice as high in lines with overexpression of *AtPDAT1* (Fig. 8F). The cultivation of the tested lines in standard conditions, despite the observed phenotypic effects and changes in lipid metabolism, did not show significant differences between WT and OE lines as in the tested plants exposed to stress conditions, in relation to any of the examined autophagy markers – their protein levels and relative expression (Supplemental Fig. S8). Only significant difference indicating increased autophagy efficiency in *AtPDAT1*-overexpressing lines grown in standard conditions was noted for the relation of lipidated and free forms of ATG8. Both structures of this protein were observed by separating protein extracts on 6 M urea gel and further incubating the blots with anti-ATG8a antibodies. In case of overexpression lines, ATG8 conjugated with PE the sum of the two forms of ATG8, constituted 78% (OE1) and 81% (OE2), whereas for control lines it was about 71% (Fig. 9AC). Both forms of ATG8 were barely detectable for all tested lines exposed to cold, indicating a significantly reduced intensity of autophagy, since the presence of lipidated form of ATG8 directly implies efficiency of autophagosome formation (Fig. 9B).

**Table 2:** The duration of individual stages of Arabidopsis life cycle subjected to cold stress. Data indicate corresponding points of development - appearance of flower bud, first flower open or first yellow leaf - were calculated based on the date of the plants sowing. Means and standard deviations were calculated from at least seven biological replicates. (^a^) denotes significant differences compared to WT in two-tailed Student’s t-test at p < 0.001

Plant line	Appearance of flower bud	First flower open	First yellow leaf
**WT control**	28.9 ± 4.1	47.3 ± 3.3	54.2 ± 0.6
**OE1**	21.4^a^ ± 1.8	27.1^a^ ± 2.8	69.6^a^ ± 5.1
**OE2**	19.8^a^ ± 1.3	24.6^a^ ± 0.8	71.1^a^ ± 5.0

## Discussion

### *PDAT1* overexpression accelerates growth rate at early and vegetative stages of development

*PDAT*-overexpressing lines exhibited a clear phenotype manifested by faster development of plants from germination to flowering and emergence of first siliques. Likewise, measured biomass content during these periods was about 50% higher than for control plants. These observations regarding the growth rate are in-line with previous results [7], conducted on in vitro cultivated seedlings. In case of mass measurement, above mentioned scientists showed that dry weight of mature plants, cultivated in growth chambers, was slightly elevated for *PDAT* overexpression. In this study we noticed the same correlations. From about eight weeks of plant cultivation in standard conditions differences in height and speed of development diminished. Cultivation in cold conditions (6 ^o^C) leads to similar observation. Even though the low temperature affected overall plant development time, the emergence time of first flower and further generative development of overexpressing lines was extremely accelerated. These observations also manifested as elevated levels of biomass measured at given time points, reaching even triple mass of control plant, which correlates with our studies done for in vitro cultures [13]. The dry biomass of these mature overexpressing lines was not so exceptional but still at least 50% higher than in standard conditions. These results suggest that PDAT enzymes might play a more important role in plant development at the early stages of growing and initiation of generative organs. Till now, for yeast, green alga and Arabidopsis leaves it was shown that PDAT enzymes plays important role in TAG synthesis during cell division and growth [26–28]. Fan et al. [28] also showed that in growing Arabidopsis leaves *PDAT1* is much expressed compared with senescing leaves. Naturally elevated expression levels of this gene were observed in wild-type plant of *A. thaliana* and *C. sativa* subjected to stress conditions like heat and cold, respectively [12, 13], which directly indicates essential role of PDAT in adverse temperature conditions and may clarify why *AtPDAT1* overexpressing lines may maintain better vigor during thermal stress.

### *PDAT1* overexpression and *SDP1* synergistic effect boost biomass increment of overexpressing lines cultivated in cold

Previously, we showed that *AtPDAT*-overexpressing lines exhibited high PDAT activity and elevated ability to synthetize triacylglycerol [13]. Observed activity also correlates with high expression level of PDAT, determined in this study. Despite this evidence, no changes in TAG content were detected, which was also previously observed by Ståhl et al. [10] and Demski et al. [13]. Tremendous increase in biomass, high *PDAT* expression levels and confirmed elevated activity with no change in the TAG pool prompted us to verify possible subsequent pathway of the TAG pool such as hydrolysis and lipid turnover. Unlike animals, plants can convert fats into one of the basic building blocks – glucose, which occurs through a series of reactions like β-oxidation, glyoxylic cycle, and subsequent gluconeogenesis. Prior to FA breakdown in β-oxidation process occurring in peroxisomes, they must be first released from the TAG pool [29, 30]. One of the TAG lipases - SDP1 (SUGAR-DEPENDENT lipase) is not only active in developing seeds but also in leaves [20, 22, 31]. Our analysis of *SDP1* expression levels exhibited strong correlation between overexpressed *PDAT* gene and elevated expression of *SDP1.* This synergistic effect was observed for plants cultivated in both tested conditions. This finding is in-line with the study carried by Fan et al. [22], where they showed that overexpression of *PDAT* directly affect *SDP1* and suggested that both enzymes drive FA β-oxidation *via* TAG intermediates. In our study we did not observe changes in *SDP1* expression levels in control plants cultivated in these conditions. This change was exclusively noted for *AtPDAT1*-overexpressing lines, which also supports the conclusion that PDAT elevated activity can stimulate SDP1. As it was already proven, mobilization of TAG pool and activity of SDP1 are connected with PXA1 (PEROXISOMAL TRANSPORTER1) responsible for FA transport to peroxisomes [22]. Therefore, PDAT and SDP1 joint activities drive the TAG breakdown further along with conversion of fat into sugar and parallel energy production. Sugar plays a general role in plant development; its elevated level promotes its transport to the vegetative tissues fueling increased growth and development [32, 33]. As described above, *AtPDAT1*-overexpressing lines tested by us exhibited phenotype characterized by huge increase of biomass. Based on our identification of elevated expression of *SDP1* and previous results we can postulate that PDAT in leaves stimulates not only TAG production but also its further metabolism and conversion into simple substances utilized directly for plant growth. Even more note-worthy was the biomass increase in *AtPDAT1*-overexpressing lines subjected to cold, which might have been the effect of stronger PDAT and SDP1 activity (which increased expression levels were identified).

Despite indicated TAG breakdown, the content and composition of the TAG pool in leaves did not change. Study done by Fan et al. [28] postulated that overexpression of PDAT stimulated fatty acid synthesis (FAS), which was confirmed by pulse-chase experiments with ^14^C-acetate. They also noted that the lack of changes in lipid content must have been the consequence of constant lipid turnover. Their results are in-line with our observations – detection of no difference in lipid content and composition. Our examination of phospholipid remodeling present in leaves supports lipid turnover hypothesis. Recent studies have already revealed tight correlation between membrane lipid and TAG metabolism in yeast and plants [28, 34]. For TAG biosynthesis PDAT enzymes utilize polyunsaturated or oxygenated acyl groups from phospholipids, especially from phosphatidylcholine and phosphatidylethanolamine, which are mainly conjugated in sn-2 position of the aforementioned phospholipids [2, 3, 10]. Utilization of the PC and PE leads to production of not only the TAG pool but also the by-products: lysophospholipids – lysophosphatidylcholine and lysophospahtidylethanolamine, respectively. In turn, lysophospholipids, are a substrate for LPLAT reactions in respective phospholipid syntheses [17, 35]. This reaction also needs acyl-CoA and in this way decreases its cytoplasmic levels, which could entail additional biosynthesis of fatty acids. Thus, PDAT enzymes actively, but not directly participate in phospholipid remodeling, and increased *de no*vo fatty acids biosynthesis, as it was shown in in vitro experiments by Fan et al. [28]. Remodeling of PE was primarily strengthened in OE lines cultivated in standard conditions, while for PC this process was highly effective in both conditions. Increased activities of LPCAT and LPEAT were also identified and shown to have improved activities in microsomal fractions derived from leaves of overexpression lines. Phospholipids play a key role, not only as a FA supplier for TAG, but also in maintenance of proper structure and fluidity of the membrane. Greater efficacy of phospholipid remodeling process is demanded to regulate membrane fluidity and to purify membrane from oxygenated fatty acids. In turn it protects the membrane from lipotoxicity and negative effects of oxidative stress during plant development and their cultivation in adverse abiotic conditions, which ultimately affects maintenance of the greater plant fitness and may additionally explain the observed improved *AtPDAT1*-overexpressing lines growth.

### Potential role of *AtPDAT1* overexpression boosting seed yield of plants cultivated in cold

Despite general detrimental effects of cold on crops, seed yield and content of acyl-lipids in seeds of *AtPDAT1*-overexpressing lines subjected to long-term cold were significantly elevated. Acyl-lipid content constitutes on average 1716 nmol of fatty acids in acyl lipids/mg seeds of OE lines in comparison to 1262 nmol in mg of control seeds, while fatty acid composition did not deviate significantly from composition of control seeds and seeds of tested lines cultivated in standard conditions. Current data strongly emphasize the problem connected with yield loses caused by plant cultivation at low temperatures [36–38]. Also, in our study we noticed seed yield reduction of control plants subjected to cold (about 15%). Nevertheless, OE lines were able to produce greater seed yield, which despite the adverse temperature conditions was even about 16–19% higher than the yield obtained from these lines from standard conditions. The differences in oil production was even greater. OE lines produced about 150% more oil in seeds/plant compared to control plants in standard conditions and about 280% in cold. At low temperatures OE lines also produced about 160% of oil produced by these lines in standard growth conditions. However, it should be noted that also another acyltransferase – DGAT, is involved in TAG biosynthesis in Arabidopsis [8]. Thus, we cannot exclude that observed effects were the results of simultaneously increased activities of DGAT type of enzymes as well. Nevertheless, we postulate, based on our observations, a promising, unknown up until now, role of PDAT as a booster for increased production of oils in oilseed plants cultivated at low temperatures.

### Autophagy flux stimulation and longevity is promoted via *AtPDAT1* overexpression

Even though *AtPDAT*1-overexpressing lines exhibited accelerated development and growth rate, our observations indicated that the beginning of senescence was postponed. The appearance of the first yellow leaf is generally recognized as a first sign of this process [39]. We noticed delayed emergence of this sign for *AtPDAT1*-overexpressing lines cultivated in standard as well as in cold conditions [13; present study]. These observations prompted us to investigate the autophagy process intensity, which is directly related to plant maturation, maintenance of fitness and longevity. For this purpose, we evaluated the expression and protein levels of two autophagy markers – NBR1 and Atg8. Atg8 protein levels were examined with application of non-denaturing and denaturing conditions. The first one provided us with data about the total amount of Atg8 protein and its intermediates/isoforms. Till now they were directly identified only in yeast [40, 41], but their occurrence is also postulated in plants [25, 42]. Whereas separation in denaturing conditions allowed us to measure the amounts of both the present lipidated (conjugated with PE), and free forms of Atg8, which are considered as direct indicators of autophagy flux intensity as they participate in final proper formation of autophagosome vesicles [43–45]. Examination of NBR1 and total Atg8 protein levels in plants grown in standard conditions did not show any significant differences between control and *AtPDAT1*-overexpressing lines. Similar results were noticed in our previous study done for 3-week-old seedlings cultivated in vitro in optimal conditions, where *Atg8* expression levels were detected [13]. In the presented study, only analysis of lipidated forms of Atg8 showed that *AtPDAT1*-overexpressing plants might have performed a more efficient autophagy process, as indicated by the results displaying higher abundance of Atg8-PE forms. Mizushima et al. [43] already described that the amount of this form correlates with autophagic activity in a mammalian cell. Further study done on Arabidopsis also proved this correlation in plants [44]. Autophagy plays an essential role in plant growth and development in optimal conditions, and its enhanced activity may better affect plant fitness and prolong its lifespan, which we observed in our tested lines. This process is also extremely important in order to cope with various stresses e.g. cold, which we examined in our study. Our first analysis, including the comparison of marker protein levels in both cold and standard conditions, revealed that autophagy process was weakened and/or slowed down. In case of NBR1, which is a selective autophagy receptor recognizing aggregated protein and pathogens [25, 46], its levels were considerably higher for plants cultivated in cold. Atg8 protein levels, which participates in autophagosome biogenesis, were diminished. Despite the data showing that autophagy was extremely low in cold conditions, *AtPDAT1*-overexpressing lines still exhibited enhanced autophagy intensity in comparison to wild-type. The levels of NBR1 protein were reduced and Atg8 protein levels were upregulated, which was supported by their expression levels. To summarize, NBR1 protein was degraded as a consequence of the ongoing autophagy process, so its levels were higher when this process was decreased and the protein itself was not metabolized [25, 46]. The opposite results were observed for Atg8, which increases during active autophagy. Atg8 present at the outer membrane of autophagosome is not degraded and can be recycled [25, 44, 47]. Thus, even if autophagy process in cold conditions is diminished compared to standard condition, in *AtPDAT1*-overexpressing lines it is still higher than in control plants and could be responsible, at least partially, for greater fitness and longevity of these overexpressing lines subjected to such adverse environment. This hypothesis cannot be supported by measurement of Atg8-PE levels which play a key role in canonical autophagy and formation of autophagosome. Due to reduced autophagy and despite the attempts to separate higher amounts of proteins on SDS-PAGE gel with denaturing conditions, the precise analysis of lipidated and free Atg8 form was impossible to perform. Only negligible visible bands were observed, which additionally confirmed weakened autophagy for plant lines subjected to cold. Nevertheless, it is worth to underline that in addition to autophagic role, Atg8 plays a non-autophagic role in plants [45]. This protein may interact with ABS3 (ABNORMAL SHOOT 3), which is independent of autophagy function of Atg8, but essential for controlling plant senescence. Jia et al., [45] also postulated that during non-autophagic pathway Atg8 was directly bound to ABS3 and promoted its degradation along with late endosome and multivesicular bodies where this protein is co-localized. Therefore, the autophagy studies carried out on Atg8 protein level basis must be evaluated with some caution. Only NBR1 levels and content of the lipidated form of Atg8 are a direct indicators of autophagy efficiency. Nevertheless, despite so far not fully understood role of Atg8 in plant cells, our data strongly identified *PDAT* overexpression as an enhancer of autophagy flux efficiency and a promoter of the longevity of OE plants. PE is an essential phospholipid linked with autophagy process [42, 48]. The content of PE pool did not undergo any significant changes in examined lines despite its preferential utilization in reaction catalyzed by PDAT [10]. This could be explained by the elevated activity of LPEAT responsible for its re-synthesis. Since PE plays a substantial role in autophagy process its amount needs to remain at a stable level, especially as it is degraded, as a part of the formed Atg8-PE complexes, localized on the inner autophagosome membrane [49–51]. Thus, not only PE re-synthesis but also *de novo* synthesis could be enhanced to keep the PE amount stable. The last process, however, was not verified in this study. Previously it has been suggested that disruption of autophagy impedes membrane lipid turnover and mobilization of fatty acids from membrane lipids to TAG [52]. Fan et al. [52] also indicated that one of the autophagy varieties, the lipophagy, which requires the same core component as the macroautophagic machinery, plays the most essential role in this process. Nevertheless, the regulation and physiological function of lipophagy remains unknown. Based on our results determining the content of Atg8 conjugated with PE, mainly in standard conditions, we can suggest that this type of autophagy might be regulated also via PDAT activity. Therefore, the visible biomass increase of the overexpressing lines cultivated in standard conditions can be the result of this process, especially since lipophagy together with SDP1 lipase mediate lipid droplets’ breakdown into fatty acids, which are later utilized in β-oxidation [52]. Whereas in cold-stress conditions the intensified effects of biomass increase can be the result of the parallel activity of both lipophagy and heightened lipid turnover, processes which do not exclude each other, but rather act synergistically.

## Conclusions

Our data present strong evidence that *AtPDAT1* overexpression acts as an enhancer of autophagy flux efficiency and a promoter of the longevity of plants cultivated in cold. Arabidopsis mutant plants are characterized by accelerated generative development, increased biomass content and greater seed yield, considering both weight and acyl-lipid content. These findings provide new, valuable knowledge about the role of the PDAT enzyme in vegetative organs and its role in plant physiology, mainly in adaptation to cold growth conditions. The key point of the study is also the discovery of increased oil production in the seeds of *AtPDAT1*-overexpressing plants, not only cultivated in standard conditions, but also subjected to unfavorable conditions. Due to current, global problem with yield loses and proper plant development in harsh conditions, these results could be potentially used both for screening and for modifying plants to obtain more resistant and high-yielding plant cultivars.

## Experimental procedures

### Plant material and growth conditions

In conducted studies *Arabidopsis thaliana* ecotype Columbia 0 plant was used as the basic line. Two *AtPDAT1*-overexpresssing lines (At5g13640) were designed and described previously by Ståhl et al. [10] and Banaś et al. [13], who kindly shared both lines. As the control plant, two lines were used in all experiments: wild-type and null-segregant line derived from progeny of a transformed plant that did not inherit an incorporated transcript. The results of the control lines in all analysis (except Western blot) are presented as the mean values of both lines (no significant differences between both lines were noted).

Before sowing in soil, seeds of tested lines were vernalized 2 days at 4 ^o^C. During the experiment, plants were grown in a growth chamber under two types of conditions: standard conditions with a constant temperature of 22 ^o^C ± 1 ^o^C with 60% of relative humidity and cold condition with temperature set to 6 ^o^C ± 1 ^o^C with humidity about 80%. In both cases, plants grew in long-day photoperiod – 16 h of day (120 µmol photons m^− 2^ s^− 1^) and 8 h of night. Batch of plants subjected to long-term low temperature (until the end of the development cycle) for the first three weeks were grown in standard conditions.

To measure the biomass content aerial parts of plant cultivated in standard conditions were collected after 3.5, 4, 5 and 8 weeks from sowing and after 4, 11 and 18 weeks after transfer to 6 ^o^C for experiment testing effect of cold exposure. Aerial parts and seeds were also collected from mature plants. In case of lipid analysis, isolation of microsomal fraction and collection samples for RNA isolation and protein extraction leaves of 4 weeks old plant cultivated in standard conditions and 7 weeks old subjected to cold (including 3 weeks cultivation in standard) were used.

To test the germination efficiency of seeds harvested from plants exposed to cold, the seeds were sown on MS media containing: 0.33 x Murashige-Skoog medium, 1% sucrose and 1% agar. The sterilization and vernalization of seeds were carried out according to the method described by Demski et al. [13]. Plates with seeds were cultivated in standard conditions (22 ^o^C ± 1 ^o^C; 16 h light (120 µmol photons m^− 2^ s^− 1^) and 8 h dark).

### Biomass measurement

Based on the phenotypic analysis and the observed changes in height and rate of plant development for *AtPDAT1*-overexpressing lines compared to the WT, the biomass measurements were done. For this purpose, the aerial part of plant growing in soil was collected at certain weeks. The aerial part of plants cultivated in standard conditions were picked up at 3.5, 4, 5 and 8 weeks, while for those from cold conditions were collected at 7, 15 and 21 weeks after sowing (including 3-weeks cultivation in standard conditions). After cultivation in both environments, the plants were also collected at the end of the development cycle to determine their dry mass content. In other cases, freshly collected material was first weighed to determine its fresh weight, then dried by 48 h at 80 ^o^C, after which the weight was measured again to determine the dry weight. Later, plants were left for next 24 h in 80 ^o^C to check that plants were completely dry. After this additional step, the plants were re-weighed.

To accurately measure the weight of seeds from one plant, each plant was wrapped underneath with foil so as not to lose any seeds and collect all of them. At the end of the developmental cycle seeds were picked up and weight.

### Lipid analysis

Changes in lipid content and composition were examined for leaves and seeds collected from the studied plant lines. Lipid extractions were performed according to modified method described by Blight and Dyer [53] with additional step of immersion of leaves in boiling water to inhibit activity of phospholipases [54]. Leaf material were firstly incubated for 20 min in boiling water, subsequently drained leaves were sonicated for 5 min in 2ml of chloroform-methanol solution (1:2; v:v). The entire volume of solvents was collected into new tubes and the leaves were poured with 2 ml chloroform-methanol solution (2:1; v:v) and sonicated by 5 min collecting again retransferring solvent into collection probes. This step was repeated twice. Collected extracts were dried under N_2_ and dissolved in 2 ml of chloroform. In case of lipid extraction from seeds, 4–5 mg of seeds were directly homogenized with addition of chloroform:methanol mixture (1:2; v:v) and 1.25 ml of 0.15 M acetic acid. To the obtained homogenates 1.25 ml of chloroform and 1.25 ml of water were added. After vigorous mixing and centrifugation, the chloroform fractions (containing lipids) were collected, dried under a N_2_ and dissolved in 1 ml of chloroform.

To determine the total content and composition of acyl-lipids’ fatty acids in tested samples, 10% of extracts were transmethylated 40 min at 90 ^o^C with addition of 2% H_2_SO_4_ in dry methanol. After incubation, 17:0-Me (internal standard) was added to the methylation mixtures. The fatty acid methyl esters were used for quantitative analysis conducted by gas chromatography GC-2010 (Shimadzu) equipped in a flame ionization detector (FID) and a 60 m×0.25 mm CP-WAX 58-CB fused-silica column (Agilent Technologies).

### Microsomal preparation and enzymes assays

Isolation of microsomal fraction from leaves of tested lines cultivated in both cold and standard conditions were conducted with accordance to method described by [55]. Rosettes of at least nine tested plant lines were firstly grided manually in glass homogenizer with addition of extraction buffer (1 mg/ml BSA, 0.33 M sucrose and 1.000 U/ml catalase dissolved in 0.1 M phosphate buffer with pH 7.2). Homogenized tissue was filtered through double Miracloth and centrifuged twice (1^st^ – 20.000 x g, 12 min; 2^nd^ – 100.000 x g, 90 min Beckman L-70 (Beckman). The resulting pellets containing microsomal fractions were washed with 0.1 M phosphate buffer (pH 7.2) and suspended in the same buffer. Aliquots of isolated fractions were used to ascertain concentration of the membrane concentration by determining phosphatidylcholine content, according to method described by Klińska et al. [55].

The microsomal fractions were used in in vitro assays to verify activity of LPEAT and LPCAT enzymes, therefore, to measure the activity of the *de novo* synthesis of PE or PC, correspondingly, when the reaction is favored into forward direction by adding exogenous LPL. To reaction mixtures 5 nmol of sn-1-18:1-lysophosholipids (LPE or LPC), 5 nmol of [^14^C]18:2-CoA, appropriate aliquots microsomal fraction, determine via optimization step, were added. Optimization assays were conducted for microsomal fraction isolated from plant subjected to cold conditions to establish best parameters for LPEAT and LPCAT activity assessment. Assays were performed in total volume of 100 µl 0.04 M phosphate buffer (pH 7.2). Reactions mixtures were incubated with continuous shaking (1250 rpm) at 30 ^o^C for time set via optimization.

The assays testing activity of remodeling process were conducted as described previously by Jasieniecka-Gazarkiewicz et al. [35] and Klińska et al. [55]. In this assay, no exogenous LPL are added. The entire LPL pool is synthesized *de novo* by reverse activity of LPLAT or/and PLA_2_ and PDAT activity and subsequently utilize in synthesis of appropriate phospholipids. Each reaction mixtures contained 10 nmol of [^14^C]18:1-CoA, 0.2 µmol of CoA and 1 mg of BSA, dissolved in final volume of 100 µl of 0.04 M phosphate buffer (pH 7.2). Reactions were started by addition of microsomal fractions containing 5 nmol of endogenous PC and conducted at 30 ^o^C for 1 h.

Both kinds of enzymatic assay were terminated by addition of 375 µl chloroform:methanol (1:2, v:v), 5 µl of glacial acetic acid, 125 µl of chloroform and 125 µl of water. The chloroform fractions were collected and separated by TLC method using polar solvent (chloroform:methanol:acetic acid:water; 90:15:10:2,5). Newly synthesized, radiolabeled phospholipids [^14^C]PE or [^14^C]PC were visualized and quantified by Instant Imager (Packard Instrument Co.).

### RNA extraction and relative expression analysis

For RNA extraction, 100 mg of flash-frozen *A. thaliana* leaves of tested plant lines, cultivated 4 weeks instandard and 4 weeks in cold (preceded by 3 weeks in standard conditions) were used (collected in appointed time described in subsection ‘Plant Material and Growth Conditions’). Total RNA extraction was conducted according to the protocol of GeneMatrix Universal RNA Purification Kit (EurX). To remove any DNA contamination, isolated RNA was treated with dsDNase (ThermoFisher Scientific) and subsequently used as a template for cDNA synthesis done with application of Maxima First Strand cDNA Synthesis Kit for RT-qPCR with dsDNase (ThermoFisher Scientific). Quality and concentration of RNA were evaluated with NanoDrop.

For relative expression analysis Maxima SYBR Green/ROX qPCR Master Mix (Thermo Fisher Scientific) were used. Prior to major experiments, optimization including determination of template concentration and linearity of primer amplification were conducted. Quantitative PCR experiments were done in accordance with derived protocol in LightCycler 480 (Roche) and data processing was conducted in LightCycler 480 Software, Version 1.5. The acquired results were analyzed employing the 2^−∆∆CT^ algorithm. Values were normalized to *ACT2* (actin 2; At3g18780), and *PP2A* (protein phosphatase 2 A; At1g69960) housekeeping genes.

Above-described method was used to measure expression level of: *PDAT1* (At5g13640), *LPCAT1* (At1g12640), *LPCAT2* (At1g63050), *LPEAT1* (At1g80950), *LPEAT2* (At2g45670), *ATG8a* (At4g16520), *NBR1* (At4g24690) and *SDP1* (At5g04040). Primers used for amplification are described in Supplementary Material (Table S1).

### Autophagy protein marker determination

Determination of protein level via western blot method began from extraction of soluble protein from leaves collected from rosettes of tested plant lines cultivated in standard and cold conditions (at weeks pointed out in above-described subsection). For isolation 100 mg of freshly harvested frozen in liquid nitrogen leaves were grinded in 100 µL of extraction buffer containing 4 M urea, 10 mM DTT, 1% (v/v) Triton X-100. Homogenized extracts were kept 30 min on ice and then centrifuged by 5 min at 13.000 rpm. Concentrations of proteins in derived supernatants were calculated based on curve of known standard concentration of BSA. Determination of the concentrations of the standard curve and the tested samples was performed by colorimetric method using BCA Protein Assay Reagent (Pierce Chemical).

For determination of lipidated form Atg8 (Atg8-PE) aliquots of 10 ug of protein were used for immunoblotting. Prior to protein separation, samples were boiled with addition of 2 x Laemmli sample buffer [56] for 10 min at 100 ^o^C. Equal amounts of chilled samples were loaded and speared on previously prepared polyacrylamide gel composed of upper stacking gel (4% polyacrylamide gel; 0.1% SDS dissolved in Tris-HCl 6.8) and bottom separated gel (15% polyacrylamide gel; 6 M urea, 0.1% SDS dissolved in Tris-HCl pH 8.8). Separated proteins from the gel were transferred on nitrocellulose membranes (Invitrogen), which were blocked overnight in 5% milk in PBST at cold temperature.

To examine the total levels of Atg8 and NBR1, respectively aliquots of 10 and 30 µg of protein were used in case of testing *A. thaliana* lines cultivated in standard conditions and 20 and 40 µg for plants subjected to long-term cold stress. Prepared samples containing appropriate amounts of proteins were supplied by 4 x NuPAGE LSD sample buffer (Invitrogen) and incubated at 65 ^o^C for 20 min. Proteins were separated on NuPAGE 4–12% Bis-Tris-Gel (Invitrogen), blotted on nitrocellulose membrane and incubated in 5% milk in TBST per night.

Next day, membranes after overnight blocking in milk solution, were incubated with corresponding primary antibodies anti-Atg8 (abcam, ab77003) or anti-NBR1 (AS142805) and subsequently with secondary antibodies conjugated with horse peroxidase (Agrisera, AS09602). The reaction was developed using BM Chemiluminescence Western Blotting Kit (Roche) and detected in Chemidoc XRS+ (Bio-Rad). Different time exposure was implemented to avoid quantification of saturated signal.

For determination of integrated density values membranes from the same exposure were chosen to compare signal for each tested line. Obtained values were presented as relative value, namely as the percentage of control lines. In case of Atg8 lipidation assessment, to compare efficiency of created forms via each tested plant lines subjected to studied conditions, percentage of each form (free and conjugated with PE) was calculated assuming that both forms constitute 100%.

### Accession numbers

Sequence data from this article can be found in the GeneBank under the following accession number: *PDAT1*, At5g13640; *ACT2*, At3g18780; *PP2A*, At1g69960, *LPCAT1*, At1g12640; *LPCAT2*, At1g63050; *LPEAT1*, At1g80950; *LPEAT2*, At2g45670; *ATG8a*, At4g21980; *NBR1*, At4g24690; *SDP1*, At5g04040.

## Supplementary Information

**Additional file 1.**

## Data Availability

The datasets used and/or analyzed during the current study are available from the corresponding author on reasonable request.
