# Supplementary material for: Phospholipid:diacylglycerol acyltransferase1-overexpression stimulates lipid turnover, oil production and fitness in cold-grown plants
Source: BMC Plant Biol. 2023 Jul 26;23:370. doi: 10.1186/s12870-023-04379-5 (PMC10369929; doi:10.1186/s12870-023-04379-5)
Supplement: Supplementary file 1 — Additional file 1. [file 12870_2023_4379_MOESM1_ESM.pdf]

Supplemental Data

Phospholipid:diacylglycerol acyltransferase1-overexpression stimulates lipid turnover, oil production and fitness in cold-grown plants

Sylwia Klińska-Bąchor[1*], Sara Kędzierska[1], Kamil Demski[2], Antoni Banaś[1]

[1] Intercollegiate Faculty of Biotechnology, University of Gdańsk and Medical University of Gdańsk, 80-307 Gdańsk, Poland

[2] Department of Plant Breeding, Swedish University of Agricultural Sciences, S-230 53 Alnarp, Sweden

[*]Correspondence author: sylwia.klinska@ug.edu.pl

Supplemental Data

Table S1. List of primers used to measure relative expression analysis of chosen genes.

Gene of Interest	Primer Orientation	Primer Sequence (5' to 3')
ACT2	forward	TGGAATCCACGAGACAACCTA
	reverse	TTCTGTGAACGATTCCTGGAC
PP2A	forward	CAGATGTGCTAAAGACGGAGC
	reverse	TGTGCAACACAGTCCTGGG
PDAT1	forward	TGGCTGCTGACTACTTTGCTC
	reverse	ACCTCTGTGTTCTGAAACGAAAG
LPCAT1	forward	ACCTCTCCTTTGGCTTCTCC
	reverse	TCCTCCTTCTTTCCACGCAT
LPCAT2	forward	AGGAAAGAGACCATCGCCTT
	reverse	CGTGAAACCCGCCATGTATT
LPEAT1	forward	TATGGGACGATGGGTTTGGGTCCTT
	reverse	ACGATAAGGAGCAGAAAACAGC
LPEAT2	forward	AGGGATGAAAGCACCGAGTTG
	reverse	ACATCAAGGAAGAATGCTGTGC
ATG8a	forward	ATGATCTTTGCTTGCTTGAAATTC
	reverse	AGCCTTCTCCACAATCACG
NBR1	forward	TGATGAGGATGGGGATGTG
	reverse	AGCAGCAGAGTTAGTGGAC
SPD1	forward	AAATGGCTTACCGGAGGAAGTT
	reverse	TGAGCCCATTCCTCATAAGTCA

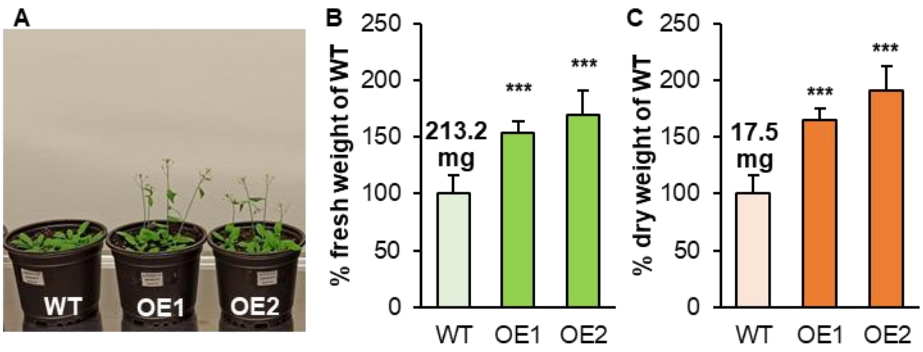

Fig. S1 Effects of 3.5-week cultivation in standard conditions on wild-type Arabidopsis (WT; control plants) and *AtPDAT1* overexpression lines (OE1 and OE2). Morphological differences between the tested lines [A]. Differences of the aerial parts in fresh [B] and dry [C] weight between WT and *AtPDAT1*-overexpressing lines. Results are presented as a percentage of control plants' weight, with mean weight written above corresponding bars. Error bars indicate standard deviation of at least seven independent biological replicates. Triple asterisks (***) demonstrate significant differences compared to WT in two-tailed Student's t-test at $p < 0.001$.

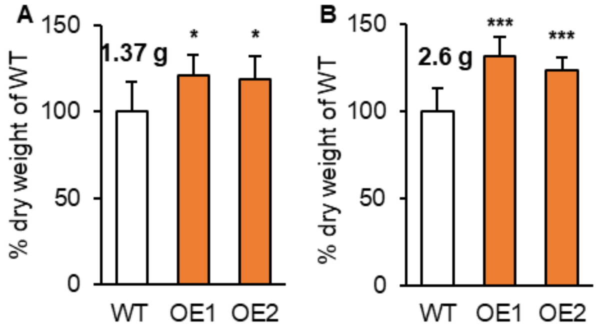

Fig. S2 Aerial parts (with seeds) dry mass of mature Arabidopsis wild-type (WT; control) and *AtPDAT1* overexpression (OE1, OE2) plants cultivated for 4 weeks in standard conditions (22 ºC), [A] and for 4 weeks in cold conditions (6 ºC), preceded by 3-week standard cultivation [B]. Results are presented as a percentage of control plants, mean values are written above corresponding bars. Error bars indicate standard deviation between of at least five independent biological replicates. Asterisks demonstrate significant difference compared to WT in two-tailed Student's t-test: (***) – $p < 0.001$; (*) – $p < 0.05$.

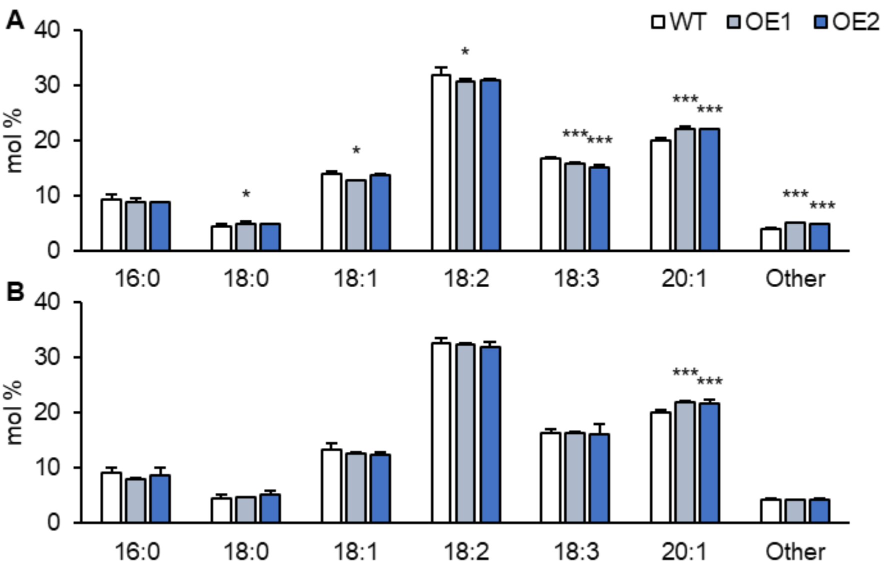

Fig. S3 Comparison of total fatty acid composition in acyl-lipids of seeds of Arabidopsis wild-type (WT; control) and *AtPDAT1* overexpression (OE1, OE2) plants cultivated for 4 weeks in standard conditions (22 ºC), [A] and for 4 weeks in cold conditions (6 ºC), preceded by 3-week standard cultivation [B]. Error bars indicate standard deviation between of three independent biological replicates. Asterisks demonstrate significant difference compared to WT in two-tailed Student's t-test: (***) – $p < 0.001$; (*) – $p < 0.05$.

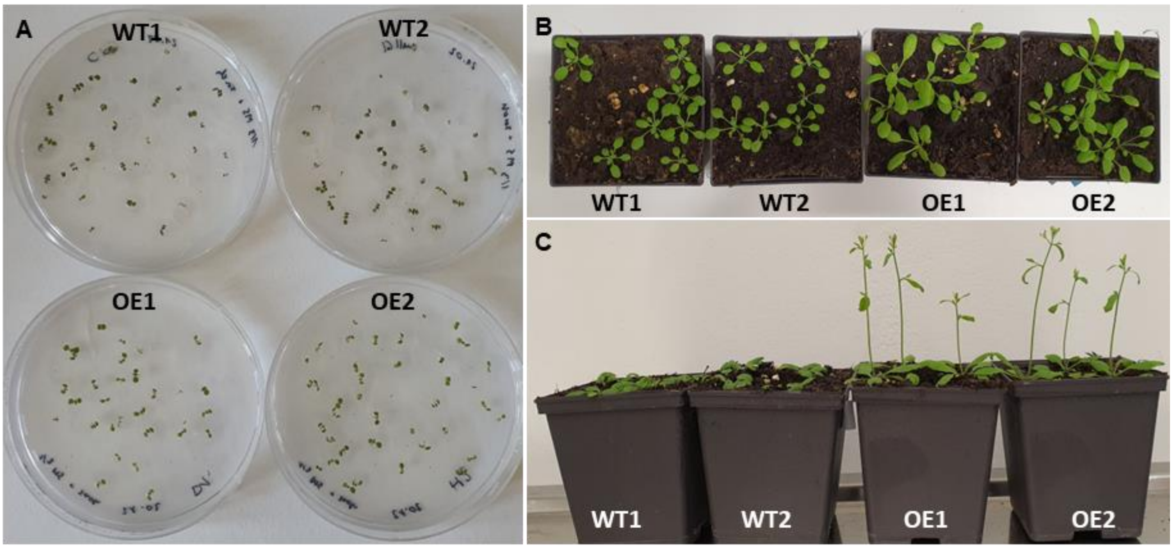

Fig. S4 Germination and further growth of Arabidopsis seeds collected from control plants (WT; control) and *AtPDAT1* overexpression lines (OE1, OE2) cultivated in cold conditions till the end of their development. Cultivation on Murashige-Skoog media in *in vitro* conditions [A] and on soil in *in vivo* conditions [B – 17 days after sowing, C – 21 days after sowing].

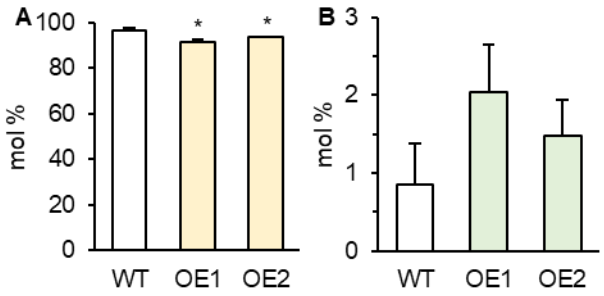

Fig. S5 Distribution of polar lipid classes [A] and triacylglycerol [B] in leaves of Arabidopsis wild-type and *AtPDAT1* overexpression lines (OE1, OE2) subjected to cold stress. Error bars indicate standard deviation between of at least two independent biological replicates. Asterisks demonstrate significant difference compared to WT in two-tailed Student’s t-test: (*) – $p < 0.05$.

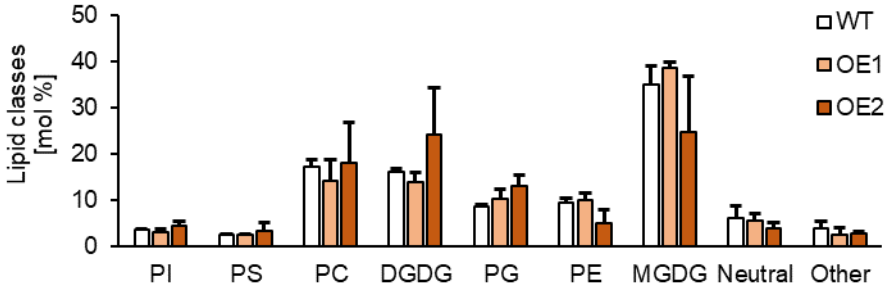

Fig. S6 Distribution of different polar lipid classes in leaves of Arabidopsis wild-type (WT; control) and *AtPDAT1* overexpression lines subjected to cold stress. Error bars indicate standard deviation between of three independent biological replicates.

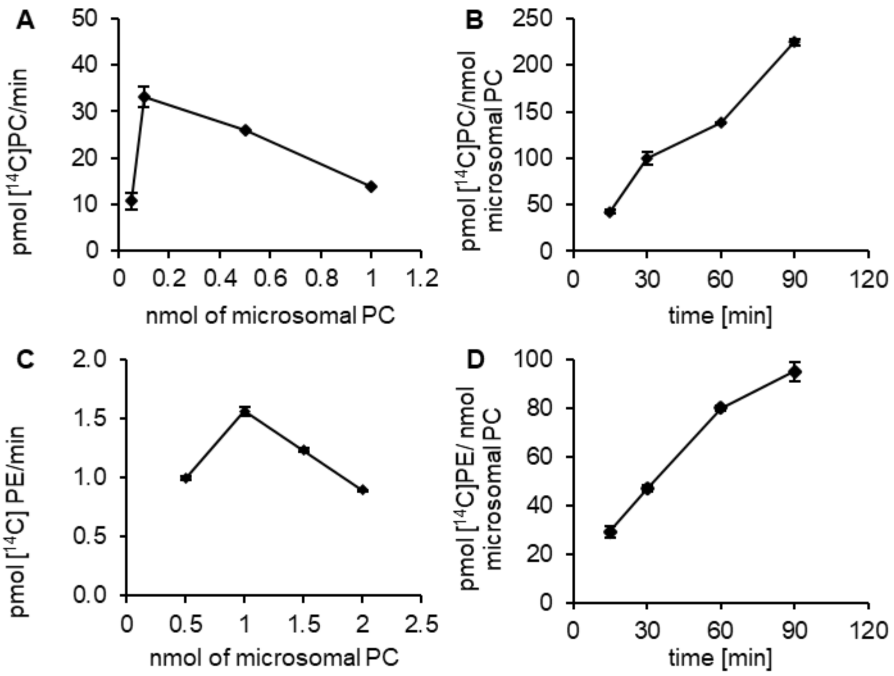

Fig. S7 Optimization of time and amount of the microsomal fraction used in *in vitro* assays analysis endogenous LPCAT and LPEAT activity. Upper graphs represent optimization results for LPCAT enzyme with regard of microsomal fraction [**A**] and reaction time [**B**]. Bottom graphs correspond to LPEAT optimization and the same parameters respectively for **C** and **D.** Error bars indicate standard deviation between of three independent replicates.

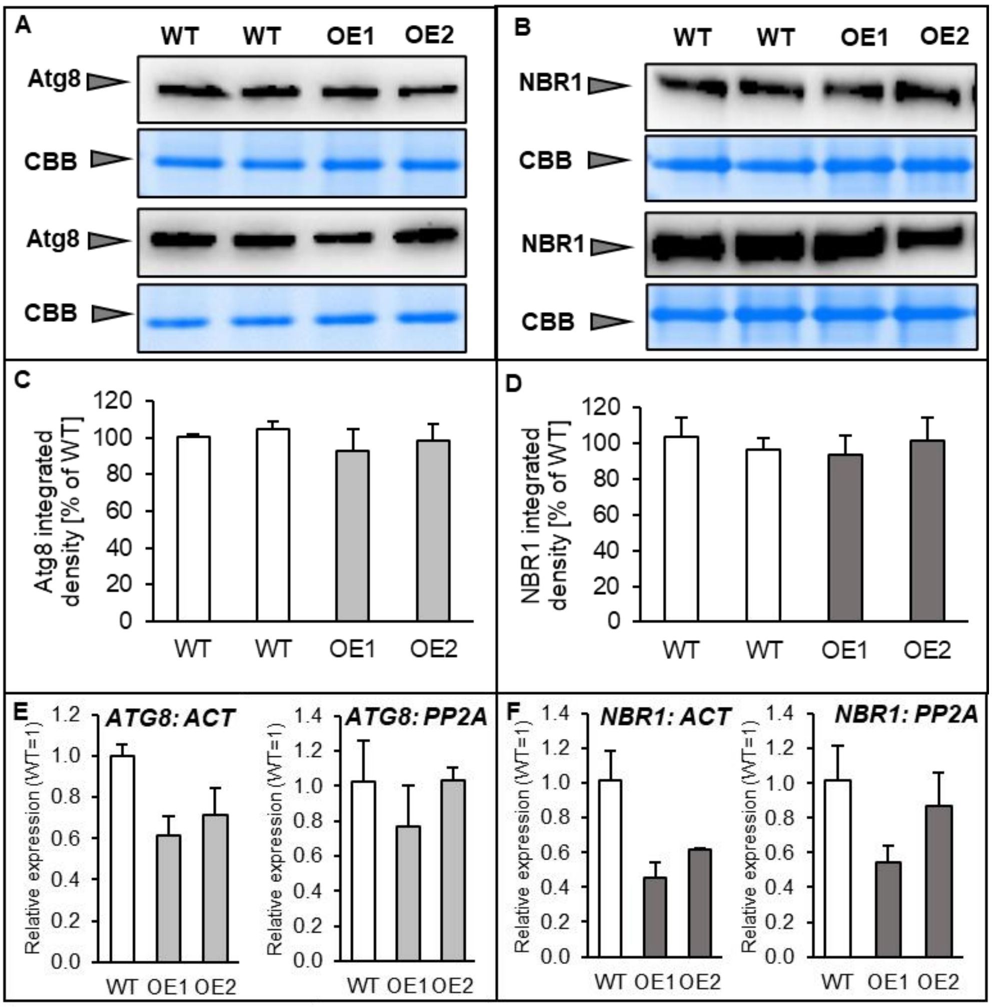

Fig. S8 Autophagic flux in Arabidopsis wild-type (WT; control plants) and *AtPDAT1* overexpression lines (OE1 and OE2) cultivated four weeks in standard conditions. Immunoblots showing endogenous ATG8 level [A] and NBR1 level [B] in leaves, after incubation with anti-Atg8 and anti-NBR1 antibodies, respectively. Coomassie blue staining were used as a loading control. Graphs **C** and **D** show quantification of bands intensity [C – Atg8 and D – NBR1]. Results are presented as a percentage of band intensity of control plants (WT). Panel **E** and **F** represent the relative expression level of genes encoding *ATG8a* and *NBR1*, respectively. Results were normalized to *ACT* and *PP2A* housekeeping genes. Above data are presents as a mean and standard deviation from at least three biological replicates. Asterisks demonstrate significant difference compared to WT in two-tailed Student's t-test: (***)

– $p < 0.001$; (**) – $p < 0.01$; (*) – $p < 0.05$. Original western blots and stained gels are shown in the Fig. S12 and Fig. S13.

Upper Blot and Gel

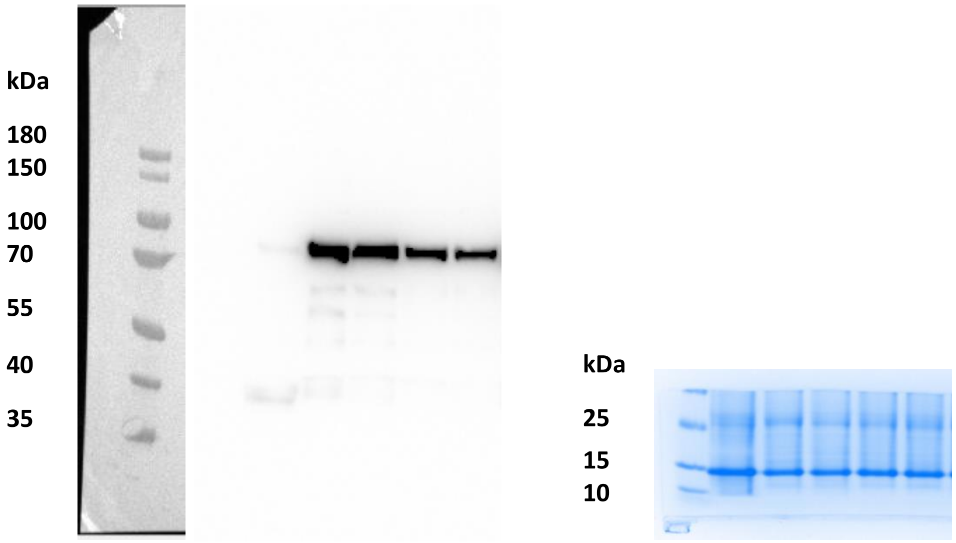

Middle Blot and Gel

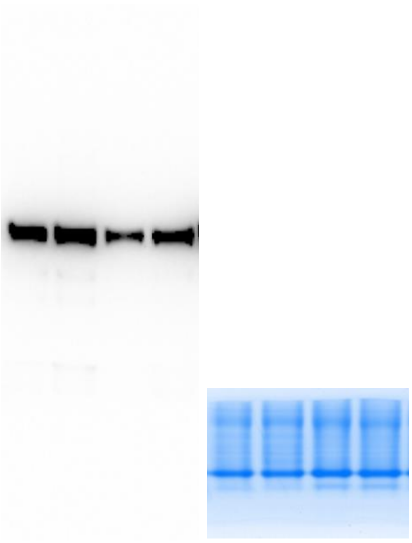

Bottom Blot and Gel

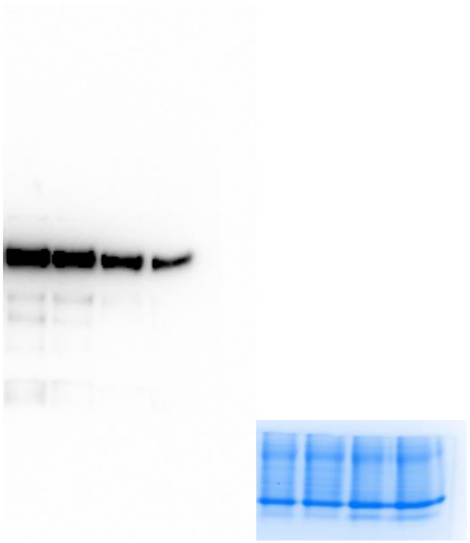

Fig. S9 Full immunoblots and gels presented in Figure 8A.

Upper Blot and Gel

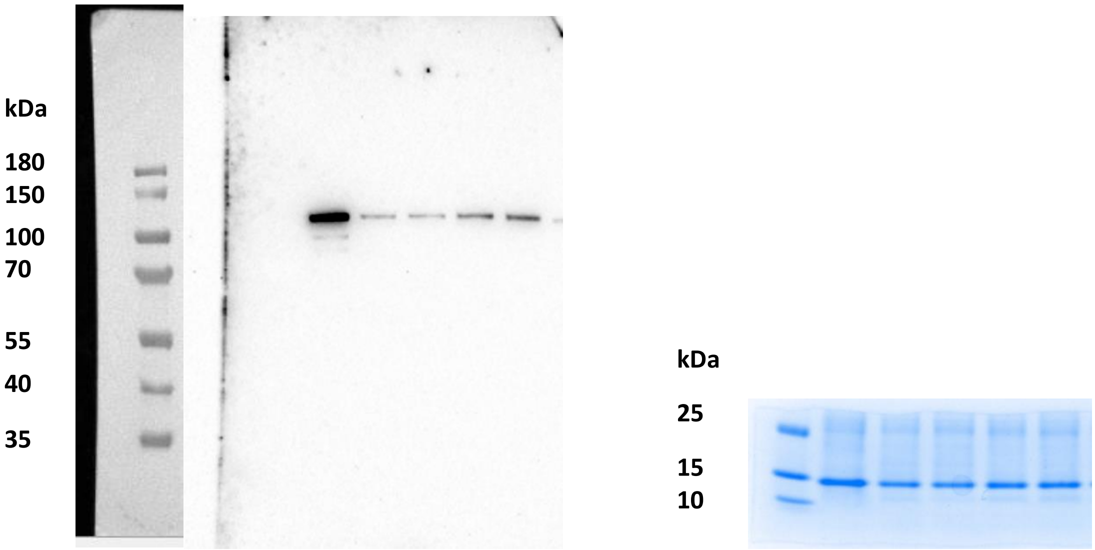

Middle Blot and Gel

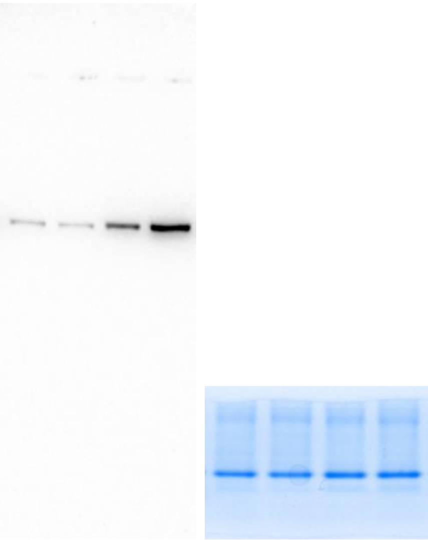

Bottom Blot and Gel

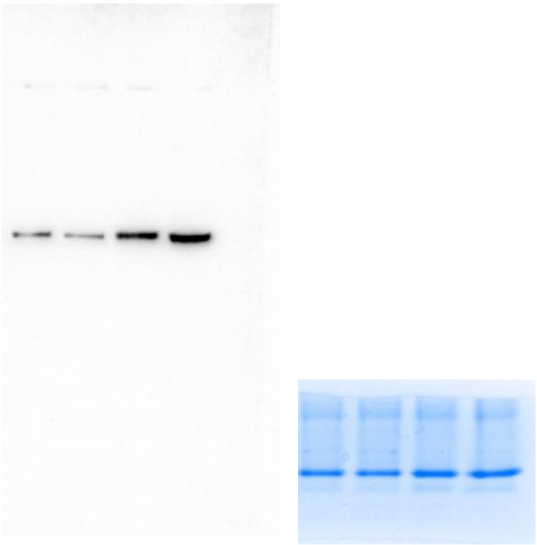

Fig. S10 Full immunoblots and gels presented in Figure 8B.

Blots and gels presented in Figure 9A

Upper Blot and Gel

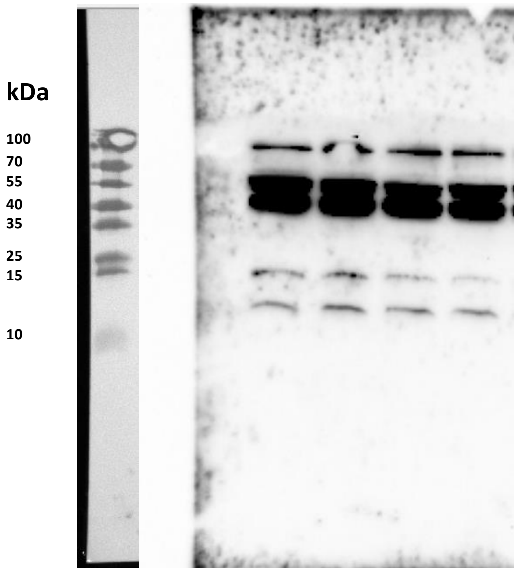

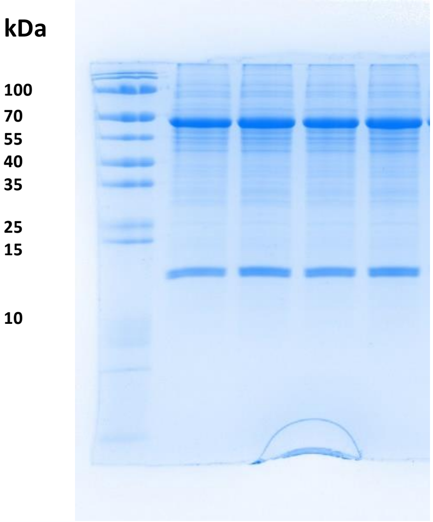

Bottom Blot and Gel

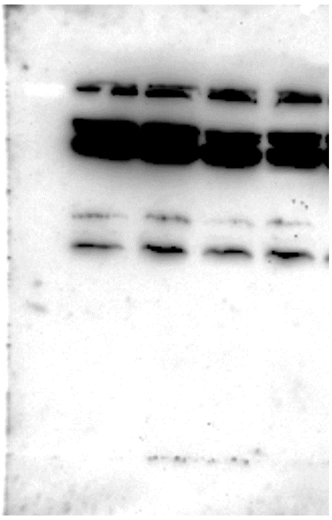

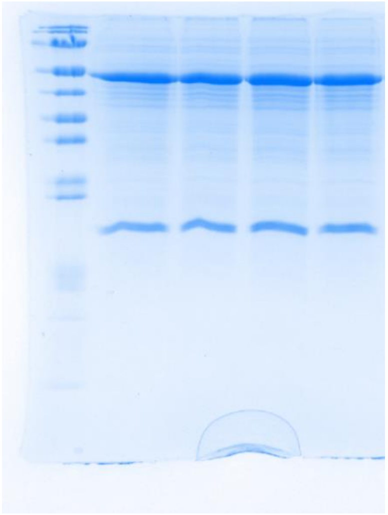

Fig. S11 Full immunoblots and gels presented in Figure 9A.

Blots and gels presented in Figure 9B

Upper Blot and Gel

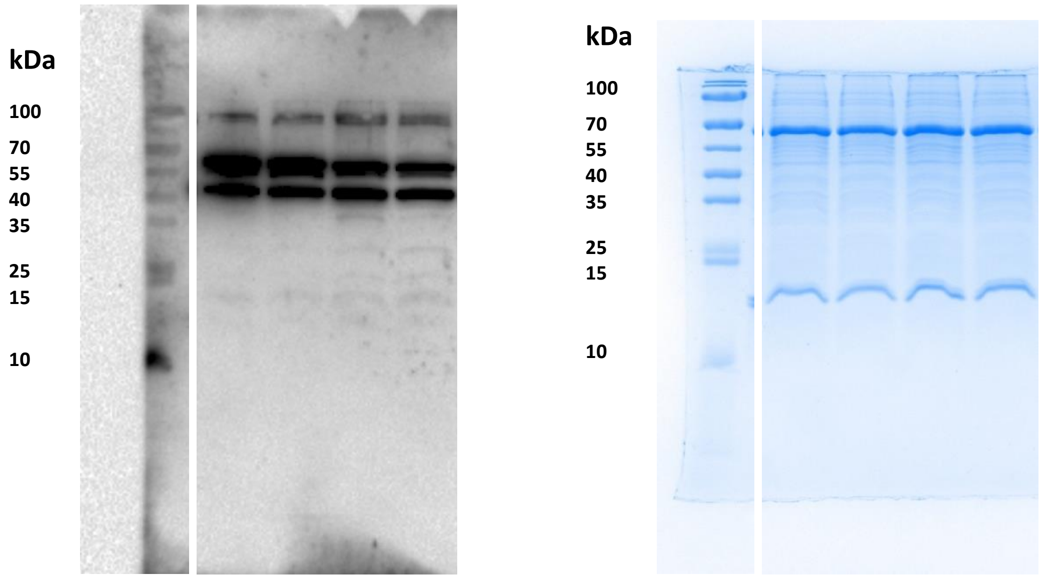

Bottom Blot and Gel

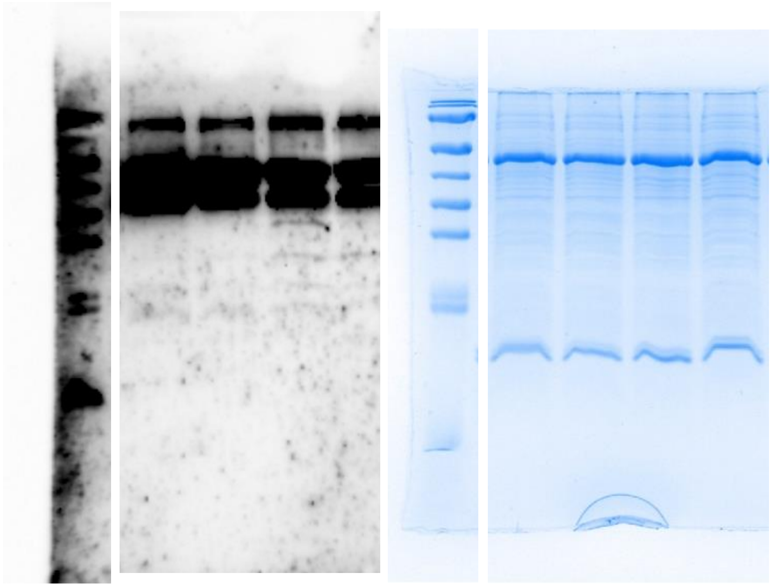

Fig. S12 Full immunoblots and gels presented in Figure 9B.

Upper Blot and Gel

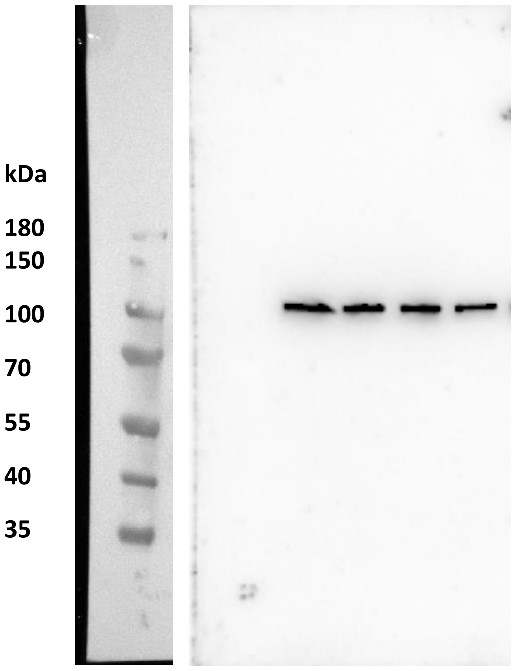

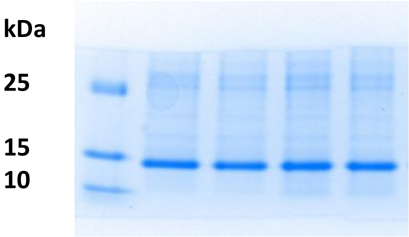

Bottom Blot and Gel

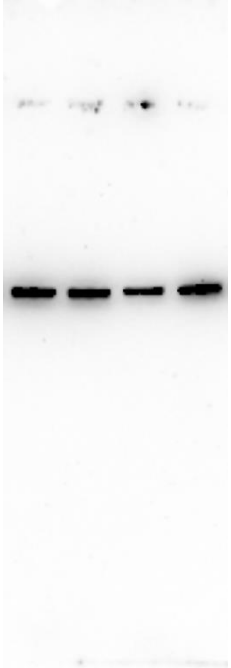

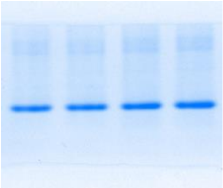

Fig. S13 Full immunoblots and gels presented in Figure S8A.

Upper Blot and Gel

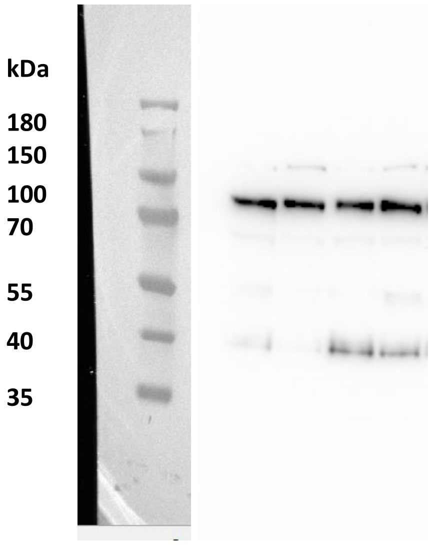

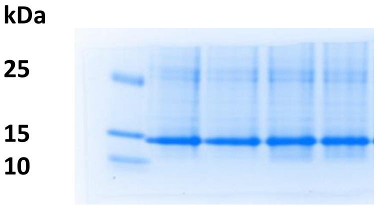

Bottom Blot and Gel

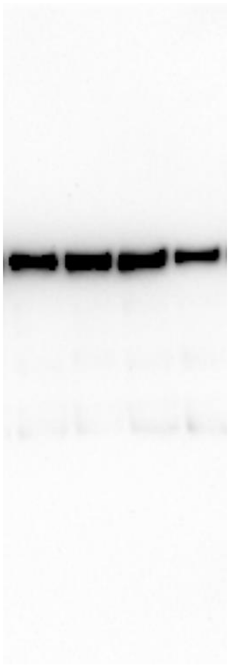

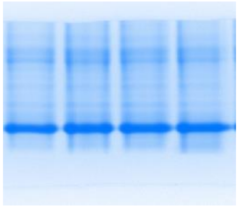

Fig. S14 Full immunoblots and gels presented in Figure S8B.
